# PI3K p85α/HIF-1α accelerates the development of pulmonary arterial hypertension by regulating fatty acid uptake and mitophagy

**DOI:** 10.1186/s10020-024-00975-9

**Published:** 2024-11-11

**Authors:** Chenyang Chen, Sirun Qin, Xiaohua Song, Juan Wen, Wei Huang, Zhe Sheng, Xiaogang Li, Yu Cao

**Affiliations:** 1 Cardiovascular Department, The Third Xiangya Hospital of Central South University, Changsha, 410013 China; 2 Department of Pediatrics, The 921, Hospital of Joint Logistics Support Force of Chinese People’s Liberation Army, Changsha, 410011 China

**Keywords:** Pulmonary arterial hypertension, Mitophagy, Fatty acid intake, HIF-1α, CD36

## Abstract

**Background:**

Pulmonary arterial hypertension (PAH) is characterized by lipid accumulation and mitochondrial dysfunction. This study was designed to investigate the effects of hypoxia-inducible factor-1α (HIF-1α) on fatty acid uptake and mitophagy in PAH.

**Methods:**

Peripheral blood samples were obtained from PAH patients. Human pulmonary arterial smooth muscle cells and rat cardiac myoblasts H9c2 were subjected to hypoxia treatment. Male *Sprague–Dawley* rats were treated with monocrotaline (MCT). Right ventricular systolic pressure (RVSP), right ventricular hypertrophy index (RVHI), pulmonary artery remodeling, and lipid accumulation were measured. Cell proliferation and ROS accumulation were assessed. Mitochondrial damage and autophagosome formation were observed. Co-immunoprecipitation was performed to verify the interaction between HIF-1α and CD36/PI3K p85α.

**Results:**

HIF-1α, CD36, Parkin, and PINK1 were upregulated in PAH samples. HIF-1α knockdown or PI3K p85α knockdown restricted the expression of HIF-1α, PI3K p85α, Parkin, PINK1, and CD36, inhibited hPASMC proliferation, promoted H9c2 cell proliferation, reduced ROS accumulation, and suppressed mitophagy. CD36 knockdown showed opposite effects to HIF-1α knockdown, which were reversed by palmitic acid. The HIF-1α activator dimethyloxalylglycine reversed the inhibitory effect of Parkin knockdown on mitophagy. In MCT-induced rats, the HIF-1α antagonist 2-methoxyestradiol (2ME) reduced RVSP, RVHI, pulmonary artery remodeling, lipid accumulation, and mitophagy. Recombinant CD36 abolished the therapeutic effect of 2ME but inhibited mitophagy. Activation of Parkin/PINK1 by salidroside (Sal) promoted mitophagy to ameliorate the pathological features of PAH-like rats, and 2ME further enhanced the therapeutic outcome of Sal.

**Conclusion:**

PI3K p85α/HIF-1α induced CD36-mediated fatty acid uptake and Parkin/PINK1-dependent mitophagy to accelerate the progression of experimental PAH.

**Graphical Abstract:**

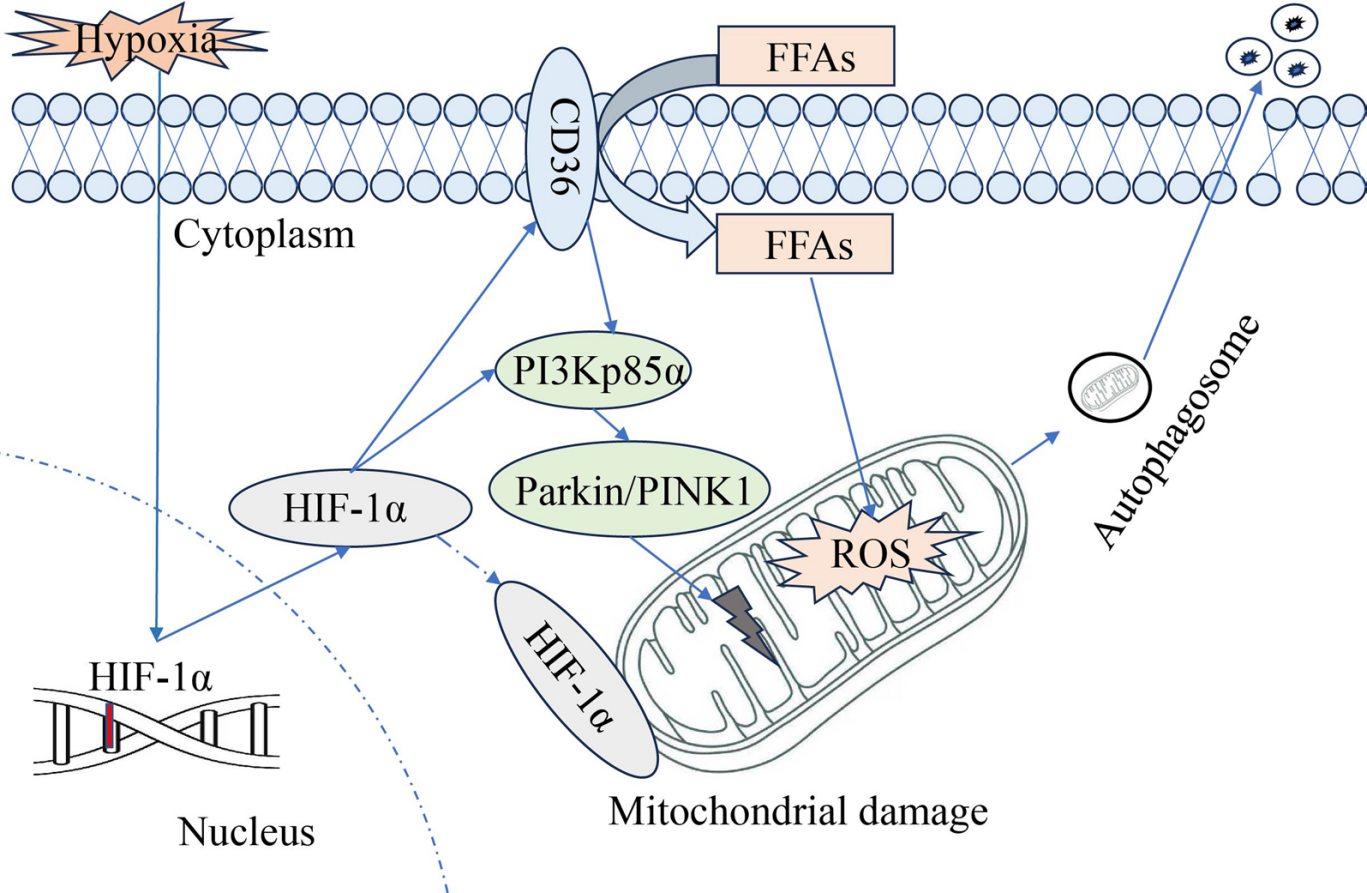

**Supplementary Information:**

The online version contains supplementary material available at 10.1186/s10020-024-00975-9.

## Introduction

Pulmonary arterial hypertension (PAH) is a chronic cardiopulmonary disease characterized by proliferation of pulmonary artery smooth muscle cells (PASMCs), which leads to a progressive increase in pulmonary vascular resistance and elevated pulmonary artery pressure, ultimately resulting in right ventricular failure and premature death (Beshay et al. 2020). Epidemiological studies have shown that the incidence of PAH is at least 15 cases per million population, with a predominance of females (Shah et al. 2022; He et al. 2022a). Despite advances in modern treatment strategies that have partially alleviated the clinical deterioration of patients, the prognosis remains unfavorable (Lau et al. 2017; Pitre et al. 2022). Therefore, understanding the pathogenesis of PAH is critical to the development of novel therapeutic approaches.

Mitochondrial dysfunction and excessive accumulation of reactive oxygen species (ROS) are considered important hallmarks of PAH (Ornatowski et al. 2020). Mitophagy, the process of removing dysfunctional mitochondria, serves to protect cells from oxidative damage and maintain mitochondrial quality (Lu et al. 2023). Under external stimuli, depolarized mitochondria accumulate phosphatase and tensin homologue (PTEN)-induced kinase 1 (PINK1) and recruit and activate the E3 ubiquitin ligase Parkin. Activated Parkin ubiquitinates proteins on the mitochondrial surface, which are then targeted to autophagosomes through the binding of the light chain 3 (LC3) receptor sequestosome-1 (p62/SQSTM1), promoting autophagic clearance of damaged mitochondria. Hypoxia, a common stimulus used to mimic PAH-like pathological conditions, leads to increased PASMC proliferation and pulmonary vascular remodeling (Shimoda 2020). Exposure to hypoxia also causes mitochondrial ROS accumulation and induces mitophagy in wild-type mice and PASMCs (Liu et al. 2022). Apoptosis-inducing factor (AIF) was found to block abnormal PASMC proliferation and pulmonary vascular remodeling by inhibiting the hypoxia-induced Parkin/PINK1 pathway and mitophagy (Ma et al. 2022). Hypoxia-inducible factor-1α (HIF-1α) has been implicated in the pathogenesis of disease during hypoxia-induced PAH (Veith et al. 2016; Luo et al. 2019). Therefore, exploring the regulatory mechanism of HIF-1α-mediated mitophagy under hypoxic conditions may contribute to the understanding of PAH.

Abnormalities in fatty acid metabolism have been observed in the heart and lungs of patients with PAH (Xu et al. 2021). Fatty acid metabolism cannot function properly without mitochondria, especially beta-oxidation. Conversely, several fatty acids and related metabolites have been characterized to influence mitophagy activity and mitochondrial function (Zhang et al. 2021). The fatty acid transporter (scavenger receptor cluster 36, CD36) is a cell surface receptor for fatty acids that allows cells to take up extracellular lipids (Wang and Li 2019). CD36 mediates oxidative stress and mitochondrial dysfunction in various cell types (Feng et al. 2024). CD36 knockdown has been shown to block PINK1/Parkin pathway activation and mitophagy in peripheral cells induced by beta-amyloid (Li et al. 2022). In addition, the crosstalk between HIF-1α, hypoxia, and fatty acid metabolism has been demonstrated. Increased expression of CD36 was found in gastric cancer cells induced by hypoxia, leading to peritoneal metastasis through the uptake of free fatty acids (Aoki et al. 2022). HIF-1α is involved in the reprogramming of fatty acid metabolism in liver cancer cells exposed to hypoxic conditions, mediating the activities of fatty acid beta-oxidation enzymes and CD36 (Matsufuji et al. 2023). However, it remains uncertain whether HIF-1α regulates CD36-mediated fatty acid uptake and mitophagy in hypoxia-induced PASMCs.

The present study demonstrated the regulation of proliferation and mitophagy in hPASMCs exposed to hypoxia, as well as in rat cardiac myoblast H9c2 cells. This regulation affected pulmonary artery remodeling and lipid accumulation and involved changes in CD36 activity as well as phosphatidylinositol 3-kinase (PI3K) p85α and Parkin/PINK1 signaling pathways. These observations helped to further elucidate the role of HIF-1α in the pathogenesis of PAH and suggested new potential strategies for the treatment of PAH.

## Materials and methods

### Patient information

A total of 20 healthy volunteers and 20 PAH patients (4 males, 16 females) were enrolled in this study. Excluding other known etiologies of PAH and severe or uncontrolled chronic diseases, 20 PAH patients included. According to different clinical and pathological features, PAH patients were classified into 18 congenital heart disease-associated PAH (PAH-01-18) and 2 idiopathic PAH (PAH-19-20). Based on the World Health Organization Functional Class (WHO-FC), the severity can be divided into grades I-IV, with grade IV being the most severe. The recruited PAH patients included 13 grade II patients and 7 grade III patients. Baseline characteristics of the subjects are shown in Supplementary Table 1. This study was approved by the IRB of Third Xiangya Hospital, Central South University (2020-S109) and in accordance with the Declaration of Helsinki, and written informed consent was obtained from each participant.

### Cell culture

Human-derived PASMCs (AW-YCH033) and H9c2 cells (AW-CNR083) were purchased from Abiowell (Changsha, China). hPASMCs were cultured in a specific smooth muscle culture system (AW-MC016, Abiowell). H9c2 cells were cultured in DMEM containing 10% fetal bovine serum (FBS) and 1% penicillin/streptomycin (P/S). All cells were grown in a 95% air, 5% CO_2_, 37 ℃ incubator. To induce hypoxia, cells were grown in a 1% O_2_, 94% N_2_, 5% CO_2_, 37 ℃ incubator (Bai et al. 2021).

### Cell grouping

Experiment 1: To investigate the effects of HIF-1α and PI3K p85α on cellular function, cells were divided into five groups: control, hypoxia, hypoxia + si-NC, hypoxia + si-HIF-1α, and hypoxia + si-PI3K p85α. Cells in the control group were grown in a normoxic environment, and the cells in the other four groups were grown in a hypoxic environment. The cells in the hypoxia + si-NC group were additionally transfected with si-NC for 24 h. The cells in the hypoxia + si-HIF-1α group were additionally transfected with si-HIF-1α for 24 h. The cells in the hypoxia + si-PI3K p85α group were additionally transfected with si-PI3K p85α for 24 h.

Experiment 2: To investigate the effects of CD36 on fatty acid uptake in cells, cells were divided into five groups: hypoxia, hypoxia + si-NC, hypoxia + si-CD36, hypoxia + PA, and hypoxia + si-CD36 + PA. In Experiment 2, cells in all groups were grown in a hypoxic environment. Cells in the hypoxia + si-NC group were additionally transfected with si-NC for 24 h. Cells in the hypoxia + si-CD36 group were additionally transfected with si-CD36 for 24 h. Cells in the hypoxia + PA group were additionally treated with 500 nM palmitic acid (PA) for 24 h (Hong et al. 2021). Cells in the hypoxia + si-CD36 + PA group were additionally transfected with si-CD36 and treated with 500 nM PA for 24 h.

Experiment 3: To investigate the effects of the Parkin/PINK1 pathway on cellular function, cells were divided into four groups: hypoxia + si-NC, hypoxia + si-Parkin, hypoxia + si-Parkin + DMOG, and hypoxia + si-PI3K p85α + DMOG. In Experiment 3, cells in all groups were grown in a hypoxic environment. Cells in the hypoxia + si-NC group were additionally transfected with si-NC for 24 h. Cells in the hypoxia + si-Parkin group were additionally transfected with si-Parkin for 24 h. Cells in the hypoxia + si-Parkin + DMOG group were additionally transfected with si-Parkin and treated with 0.1 mM dimethyloxalylglycine (DMOG, a HIF-1α activator) for 24 h (Chen et al. 2022c). Cells in the hypoxia + si-PI3K p85α + DMOG group were additionally transfected with si-PI3K p85α and treated with 0.1 mM DMOG for 24 h.

Cell transfection was performed according to the instructions of Lipofectamine 2000 reagent (11668019, Invitrogen, Carlsbad, CA, USA). All vector constructs used in cell experiments were purchased from HornorGene (Changsha, China).

### Quantitative real-time PCR (qRT-PCR)

Total RNA was isolated from lung tissue and cell lysates using Trizol (15596026, Thermo Scientific, Portsmouth, NH, USA). Subsequently, cDNA was synthesized using a cDNA synthesis kit (CW2569, CWBIO, Taizhou, China). Amplification was performed on a QuantStudio 1 Real-Time PCR system (Thermo Scientific) using the UltraSYBR Mixture reagent kit (CW2601, ConWin). The amplification program was set to 95 °C for 30 s, followed by 40 cycles of 95 °C for 5 s and 60 °C for 15 s. The primer sequences for the target genes were designed using Primer5 (PREMIER Biosoft, Vancouver, Canada) and are shown in Supplementary Table 2. The relative mRNA expression of *HIF-1α*, *PI3K*, *Parkin*, *PINK1*, *CD36*, uncoupling protein-2 (*UCP2*), manganese superoxide dismutase (*MnSOD*), unc-51-like kinase 1 (*ULK1*), B cell lymphoma 2 (BCL2)-interacting protein 3 like (*BNIP3L*), and FUN14 domain containing 1 (*FUNDC1*) was calculated using the 2^−ΔΔCt^ method, with *GAPDH* serving as the reference gene.

### 5-ethynyl-2-deoxyuridine (EdU) staining

The EdU assay kit (C10310, RiboBio, Guangzhou, China) was used to assess cell proliferation. In brief, hPASMCs and H9c2 cells after different treatments were seeded into 96-well plates at a density of 5 × 10^4^ cells per well. Diluted EDU was then added to each well and incubated overnight. Each well was sequentially incubated with 4% paraformaldehyde for 30 min, 2 mg/mL glycine for 5 min, and 0.5% TritonX-100 for 10 min. Subsequently, 1 × Apollo staining solution was added and incubated for 30 min. After washing with 0.5% TritonX-100, Hoechst33342 reaction solution was added and incubated for 30 min in the dark, followed by restaining of the nuclei with 4,6-diamidino-2-phenylindole (DAPI). Finally, positive labeling was determined by fluorescence microscopy (CX41-72C02, Olympus, Tokyo, Japan) and counted.

### Immunofluorescence (IF)

The expression of CD36, HIF-1α, and LC3 proteins in hPASMCs and H9c2 cells was assessed by IF assay. Briefly, cells were fixed by 4% paraformaldehyde and permeabilized with 0.3% Triton X-100. After cells were blocked in 5% bovine serum albumin (BSA) for 1 h, they were incubated with CD36 antibody (1: 50, 66395-1-Ig, Proteintech, Chicago, IL, USA), HIF-1α (1: 50, ab1, Abcam, Cambridge, UK), and LC3 antibody (1: 100, AWA50797, Abiowell) overnight at 4 ℃ and exposed to anti-Rabbit/Mouse IgG (H + L) (1:200, AWS0005c/AWS0004c, Abiowell). Subsequently, the nuclei were stained with DAPI and fluorescence images (magnification 400 ×) were captured with a fluorescence microscope (BA210E, Motic, Xiamen, China).

### Western blotting

Radioimmunoprecipitation (RIPA) buffer (AWB0136, Abiowell) was added to lung tissues and cell lysates and the mixture was thoroughly homogenized or disrupted to extract total proteins. The protein concentration was determined using bicinchoninic acid (BCA) assay kits (AWB0104, Abiowell). Subsequently, the proteins were separated by 10% sodium-dodecyl-sulfate–polyacrylamide gel electrophoresis (SDS-PAGE) and transferred to nitrocellulose membranes. The membranes were blocked with PBST containing 5% skimmed milk to remove nonspecific binding. The membranes were then incubated with diluted primary antibodies overnight at 4 °C, followed by incubation with diluted secondary antibodies at room temperature. The antibody information is provided in Supplementary Table 3. Finally, the membranes were exposed to ECL Plus Luminol (AWB0005, Abiowell) and imaged using a gel imaging system (ChemiScope6100, CLiNX, Shanghai, China). The protein levels of the targets were assessed by calculating ratios to the gray-scale values of GAPDH.

### Flow cytometry

ROS levels in cells and lung tissue were measured by flow cytometry. For hPASMCs and H9c2 cells, cells were detached by trypsinization and resuspended in a culture medium. For lung tissue, tissue fragments were treated with 2% collagenase for digestion and filtered to obtain cell suspension. BODIPY™ 581/591 C11 fluorescent probe (D3861, Invitrogen, Carlsbad, CA, USA) was then added to the cell suspension at a final concentration of 5 μM and incubated at 37 °C in the dark. Finally, the fluorescence intensity was detected using a flow cytometer (A00-1-1102, Beckman Coulter, Fullerton, CA, USA).

### Transmission electron microscopy (TEM)

hPASMCs and H9c2 cells, as well as lung tissues, were fixed in 2.5% glutaraldehyde (AWI0097, Abiowell) for 6–12 h and 1% osmium tetroxide (18456, TED PELLA, Inc., Redding, CA, USA) for 1–2 h. Fixed cells were dehydrated through a gradient of ethanol and propylene oxide. The cells were then immersed in a 1:1 mixture of propylene oxide and epoxy resin, followed by embedding in pure epoxy resin. After sectioning, the cells were stained with lead and uranium. Finally, the mitochondrial morphology was observed with a transmission electron microscope (JEM1400, JEOL, Tokyo, Japan).

### Co-immunoprecipitation (Co-IP)

The co-IP assay was applied to confirm the interaction of HIF-1α and PI3K p85 or HIF-1α and CD36. IP lysis buffer (AWB0144, Abiowell) was applied to lyse hPASMCs and H9c2 cells, followed by incubation with Normal rabbit IgG (B900610, Proteintech) or HIF-1α (ab179483, Abcam) antibodies overnight at 4 °C. Then, the immune complexes were mixed with Protein A/G agarose beads at 4 °C for 2 h to couple the antibodies to the agarose beads. The agarose beads were centrifuged and sank to the bottom of the centrifuge tube, and the supernatant was carefully aspirated. The agarose beads were washed with IP analysis buffer to obtain co-IP products, followed by western blotting analysis. The antibodies used for western blotting were HIF-1α (ab179483, Abcam), CD36 (18836-1-AP, Proteintech) and PI3K p85α (ab191606, Abcam).

### Animal model

Male *Sprague–Dawley* rats (230–250 g) were purchased from Hunan SJA Animal Laboratory Co., Ltd. (Changsha, China). The rats were housed in a controlled environment with a temperature of 22 ± 1 °C, humidity of 45–55%, and a 12-h light/dark cycle, and had free access to food and water. The rats were randomly divided into normal, PAH, PAH + 2ME, PAH + 2ME + CD36, PAH + Sal, and PAH + 2ME + Sal groups. To induce PAH, the rats received a single intraperitoneal injection of 65 mg/kg monocrotaline (MCT, 315-22-0, BIDE PHARMATECH Co., Ltd., Shanghai, China) (Jin et al. 2021), while the rats in the normal group received an injection of the same volume of solvent. Two days after the single injection of MCT, rats in the PAH + 2ME group received daily intraperitoneal injection of 25 mg/kg HIF-1α antagonist, 2-methoxyestradiol (2ME, 362-07-2, Macklin, Shanghai, China), for 21 days (He et al. 2022b; Azhar et al. 2020). Two days after the single injection of MCT, rats in the PAH + 2ME + CD36 group received a daily intraperitoneal injection of 25 mg/kg 2ME along with twice-weekly intraperitoneal injections of 2 μg CD36 (HY-P74303, MCE, Monmouth Junction, NJ, USA) for 21 days (Arafah et al. 2019). Two days after the single injection of MCT, rats in the PAH + Sal group received a daily intraperitoneal injection of 50 mg/kg salidroside (Sal, 10338-51-9, BIDE PHARMATECH Co., Ltd.), an activator of the Parkin/PINK1 pathway, for 21 days (Gu et al. 2020; Li and Chen 2019). Two days after the single injection of MCT, rats in the PAH + 2ME + Sal group received a daily intraperitoneal injection of 50 mg/kg Sal along with a daily intraperitoneal injection of 25 mg/kg 2ME for 21 days. Rats were euthanized by intraperitoneal injection of 200 mg/kg sodium pentobarbital. All animal procedures were approved by the Animal Welfare Ethics Committee of the Third Xiangya Hospital, Central South University (2020sydw0211).

### Measurement of right ventricular systolic pressure (RVSP) and right ventricular hypertrophy index (RVHI)

RVSP was evaluated by hemodynamic measurements (Chen et al. 2023). Rats were anesthetized with 60 mg/kg pentobarbital sodium injected intraperitoneally and fixed. The right jugular vein was exposed, and a PE-50 polyethylene catheter (SPR-320, Millar Instruments, Houston, TX, USA) was inserted through the right jugular vein into the right ventricle. The other end of the catheter was connected to a pressure transducer and the cardiovascular analysis system (Alcott Biotech, Shanghai, China). RVSP was continuously monitored in real time after pressure waveform stabilization. To measure RVHI, saline solution was infused into the left ventricle (LV) after death and the heart was removed. The right ventricle (RV) was separated from the LV and the ventricular septum (VS), and the weight of the RV and the weight of the LV plus VS were recorded. RVHI = RV/(LV + VS) (Yang et al. 2023).

### Tissue staining

Lung tissues were fixed, embedded in paraffin, and sectioned at 4–5 μm. The sections were stained with hematoxylin and eosin (AWI0020a, Abiowell), Masson’s trichrome staining kit (AWI0253a, Abiowell), and oil red O working solution (AWI0580a, Abiowell) according to the instructions of the staining kits. Finally, the sections were sealed and examined under a microscope (BA210T, Motic).

### Immunohistochemistry (IHC)

IHC assays were used to determine the expression of PCNA, Parkin, and PINK1 in lung tissue. In brief, sections were thermochemically repaired, and 1% periodate was added to eliminate endogenous peroxidase activity. After washing, sections were incubated with PCNA (1:200, 10205-2-AP, Proteintech), Parkin (1:200, AWA41194, Abiowell), or PINK1 (1:200, AWA03436, Abiowell) at 4 ℃ overnight. Then, sections were incubated with HRP goat anti-mouse/rabbit IgG (1:100, AWS0003/AWS0005, Abiowell). Hematoxylin was then used to stain the nuclei. Finally, sections were incubated in gradient alcohol (60–100%) and xylene. Finally, images were acquired by microscope (BA410T, Motic). Positive staining was quantified by Image-Pro-Plus (IPP) software.

### Enzyme-linked immunosorbent assay (ELISA)

The levels of HIF-1α, Parkin, PINK1, fatty acid synthetase (FAS), acetyl-CoA carboxylase (ACC), and CD36 in human or rat serum were measured according to the instructions of the following ELISA kits: Human HIF-1α ELISA kit (CSB-E12112h, CUSABIO Co., Ltd., Wuhan, China), Human Parkin ELISA Kit (JL11195, Shanghai Jianglai Industrial Limited by Share Ltd., Shanghai, China), Human PINK1 ELISA Kit (JL11175, Shanghai Jianglai Industrial Limited by Share Ltd.), Human/Rat FAS ELISA Kit (CSB-E04542h/CSB-E07324r, CUSABIO Co., Ltd.), Human/Rat ACC ELISA Kit (CSB-EL001119HU, CUSABIO Co., Ltd./YJ059228, Shanghai Yuanju Biotechnology Center, Shanghai, China), and Human/Rat CD36 ELISA Kit (CSB-E10117h/CSB-EL004927RA, CUSABIO Co., Ltd.).

### Statistical analysis

The data are presented as mean ± standard deviation (SD) and each experiment was repeated at least three times. Statistical analysis was performed using GraphPad Prism 9.0 (GraphPad, La Jolla, CA, USA). An unpaired Student’s *t*-test was performed when there were only two groups. Multiple group comparisons were analyzed using one-way ANOVA, followed by Tukey’s post hoc test. Spearman’s correlation analysis was performed to analyze the correlation between key indicators in the serum of PAH patients. The in vitro PASMC and H9c2 experiments included 3 biological replicates. The in vivo experiments included 6 biological replicates. For all analyses, *P* < 0.05 was considered statistically significant.

## Results

### Differential expression of HIF-1α, Parkin/PINK1 axis, and fatty acid metabolism-related factors in the serum of PAH patients

**Fig. 1:**
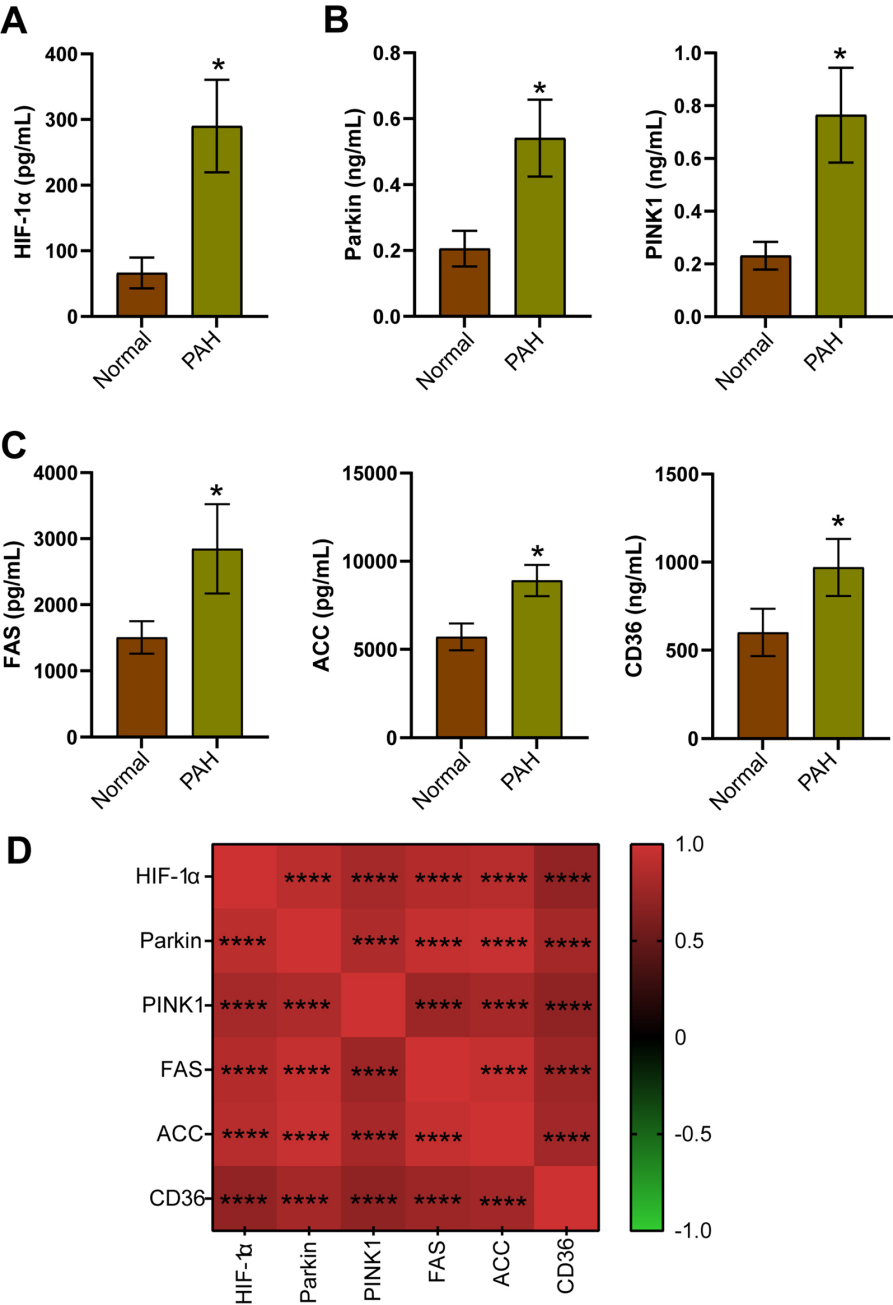
HIF-1α was closely associated with the Parkin/PINK1 axis and fatty acid metabolism-related factors in PAH patients. **A** Serum levels of HIF-1α were measured by ELISA. **B** Serum levels of Parkin and PINK1 were measured by ELISA. **C** Serum levels of FAS, ACC, and CD36 were measured by ELISA. ^*^P < 0.05 vs. Normal. The statistical test was performed using unpaired Student’s *t*-test. **D** Spearman's correlation analysis was performed to determine the correlation between HIF-1α, Parkin, PINK1, FAS, ACC, and CD36. ^*^P < 0.05. ^***^P < 0.001. ^****^P < 0.0001

The involvement of HIF-1α, Parkin/PINK1 axis, and fatty acid metabolism in the regulation of PAH and their relationships were determined. Healthy volunteers (Normal, n = 20) and PAH patients (PAH, n = 20) were included in this study. Among the PAH patients, 18 had PAH associated with congenital heart disease (PAH-01-18) and 2 had idiopathic PAH (PAH-19-20). Compared with healthy subjects, increased serum levels of HIF-1α were observed in PAH patients (Fig. 1A). The expression level of Parkin/PINK1 can indicate the level of mitophagy (Chen et al. 2024). The serum levels of Parkin and PINK1 were elevated in PAH patients (Fig. 1B). The serum levels of FAS, ACC, and CD36 were upregulated in PAH patients compared to healthy subjects (Fig. 1C). Spearman’s correlation analysis revealed a positive correlation between HIF-1α and the levels of PINK1, FAS, ACC, and CD36 (Fig. 1D). These clinical data suggested a close relationship between HIF-1α, the Parkin/PINK1 axis, and fatty acid synthesis/uptake in PAH.

### PI3K p85α/HIF-1α regulates CD36 expression, the Parkin/PINK1 pathway, and mitophagy

**Fig. 2:**
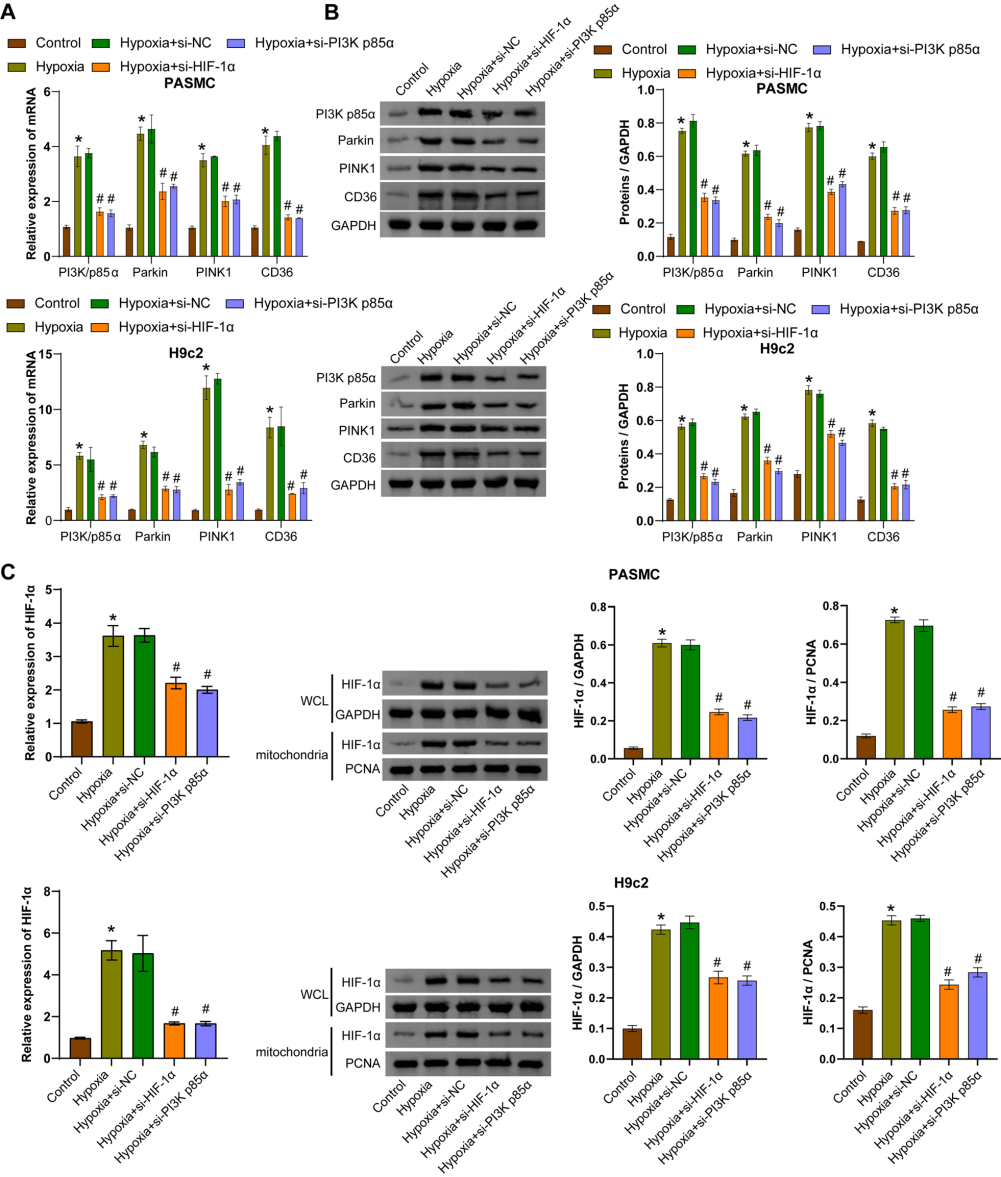

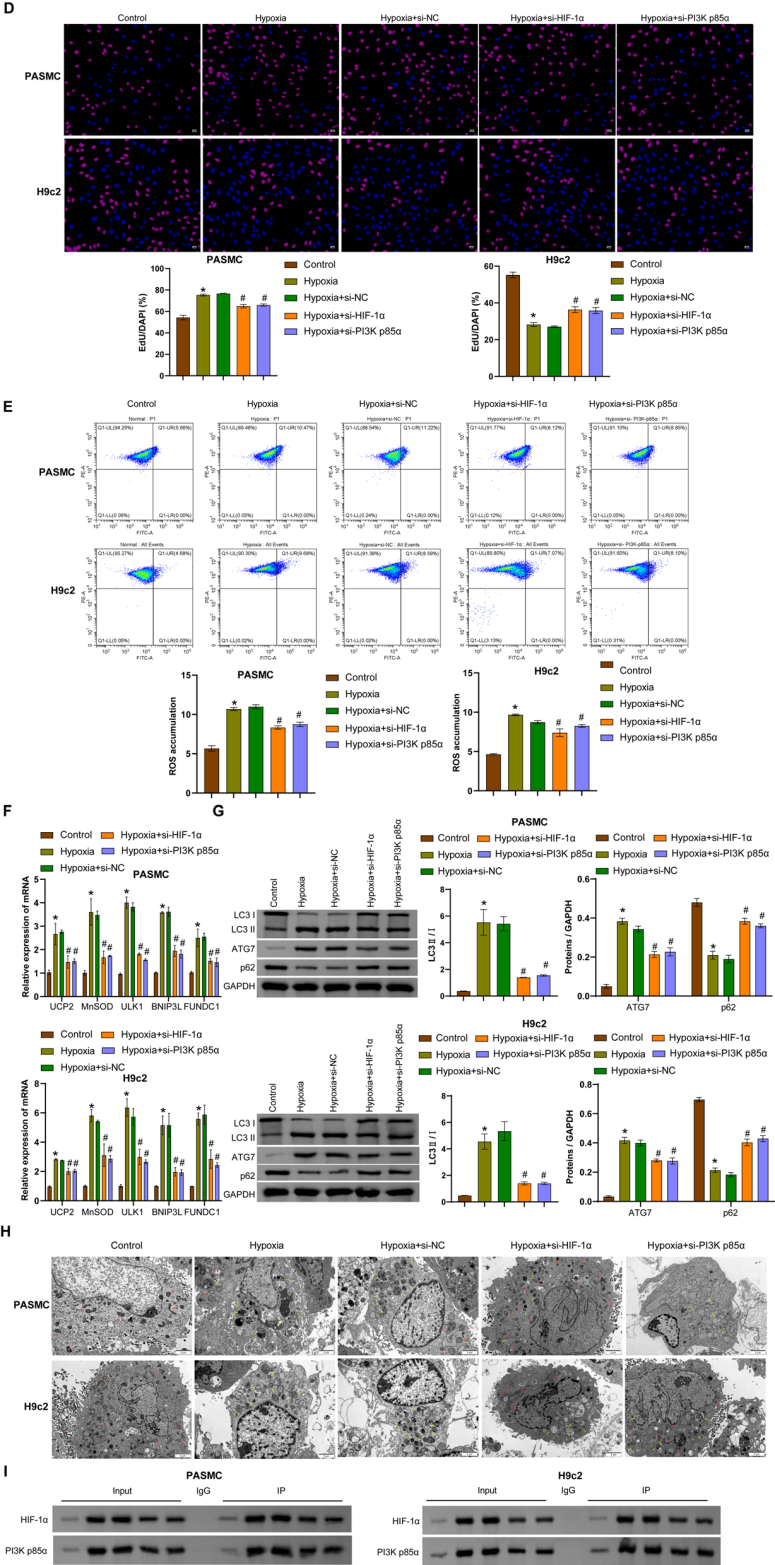
Hypoxia activated the PI3K p85α/HIF-1α axis to regulate the Parkin/PINK1 pathway, CD36 expression, and mitophagy in hPASMCs and rat H9c2 cells. **A** The mRNA levels of *PI3K p85α*, *Parkin*, *PINK1*, and *CD36* were measured by qRT-PCR. **B** Protein levels of PI3K p85α, Parkin, PINK1, and CD36 were measured by Western blotting. **C** The mRNA and protein levels of HIF-1α in whole cell lysates (WCL) and mitochondria. **D** Proliferation of hPASMCs and rat H9c2 cells was assessed by EdU staining. Scale bar = 50 μm. **E** ROS accumulation was detected by flow cytometry. **F** *UCP2*, *MnSOD*, *ULK1*, *BNIP3L,* and *FUNDC1* mRNA levels were measured by qRT-PCR. **G** Protein levels of LC3 II/I, ATG7, and p62 were measured by Western blotting. **H** Mitochondrial structure and autophagosome formation were observed by transmission electron microscopy. Autophagosomes are indicated by yellow arrows, and relatively normal mitochondria are indicated by red arrows. Scale bar = 2 μm. **I** Co-IP experiment was performed to detect the binding between HIF-1α and PI3K p85α. ^*^P < 0.05 vs. Control. ^#^*P* < 0.05 vs. Hypoxia + si-NC. n = 3. The statistical test was performed using one-way ANOVA, followed by Tukey’s post hoc test

Transfection of si-HIF-1α or si-PI3K p85α was performed in hypoxia-induced hPASMCs and rat H9c2 cells to investigate the regulatory relationship between the PI3K p85α/HIF-1α axis, the Parkin/PINK1 pathway, CD36 expression and mitophagy. Compared with the control group, the expression of PI3K p85α, Parkin, PINK1, and CD36 was increased in hypoxia-induced cells. Transfection of si-HIF-1α into hypoxia-induced cells decreased the expression of PI3K p85α, Parkin, PINK1, and CD36 compared to cells transfected with si-NC. Similarly, transfection of si-PI3K p85α into hypoxia-exposed cells resulted in decreased expression of PI3K p85α, Parkin, PINK1, and CD36 compared to cells transfected with si-NC (Fig. 2A and B). Hypoxia induced increased expression of HIF-1α in the whole cell lysates (WCL) and mitochondria compared with the control group. Transfection of si-HIF-1α or si-PI3K p85α into hypoxia-induced cells showed decreased expression of HIF-1α in WCL and mitochondria (Fig. 2C). Hypoxia-exposed hPASMCs showed increased proliferation capacity, whereas hypoxia-exposed rat H9c2 cells showed decreased proliferation capacity. Transfection of si-HIF-1α or si-PI3K p85α reversed the proliferation of hPASMC and rat H9c2 cells compared to cells transfected with si-NC (Fig. 2D and S1A). Furthermore, hypoxia-induction increased ROS levels in hPASMCs and rat H9c2 cells, whereas transfection of si-HIF-1α or si-PI3K p85α decreased ROS levels (Fig. 2E). Compared to untreated cells, hypoxia-induced levels of mitochondrial antioxidant genes *(UCP2* and *MnSOD*) and mitophagy-inducing genes (*ULK1*, *BNIP3L*, and *FUNDC1*) were increased. Transfection of si-HIF-1α or si-PI3K p85α caused downregulated levels of these factors compared to hypoxia-challenged cells transfected with si-NC (Fig. 2F). Transfection of si-HIF-1α or si-PI3K p85α also reversed the hypoxia-induced increase in LC3 II/I and ATG7 expression and the hypoxia-induced decrease in p62 expression (Fig. 2G). Mitochondrial structural changes and the number of autophagosomes were observed by TEM to determine the severity of mitophagy (Zhu et al. 2024). TEM analysis revealed increased damaged mitochondria and autophagosomes in hypoxia-treated cells, whereas transfection of si-HIF-1α or si-PI3K p85α reduced the number of damaged mitochondria and autophagosomes (Fig. 2H). Co-IP analysis revealed an interaction between HIF-1α and PI3K p85α (Fig. 2I). These results suggested that HIF-1α/PI3K p85α regulated CD36 expression and ubiquitin-dependent or -independent mitophagy in hPASMCs and rat H9c2 cells under hypoxic conditions.

### Hypoxia activates the HIF-1α/CD36 *axis* to regulate fatty acid uptake, autophagy-related markers, and Parkin/PINK1

**Fig. 3:**
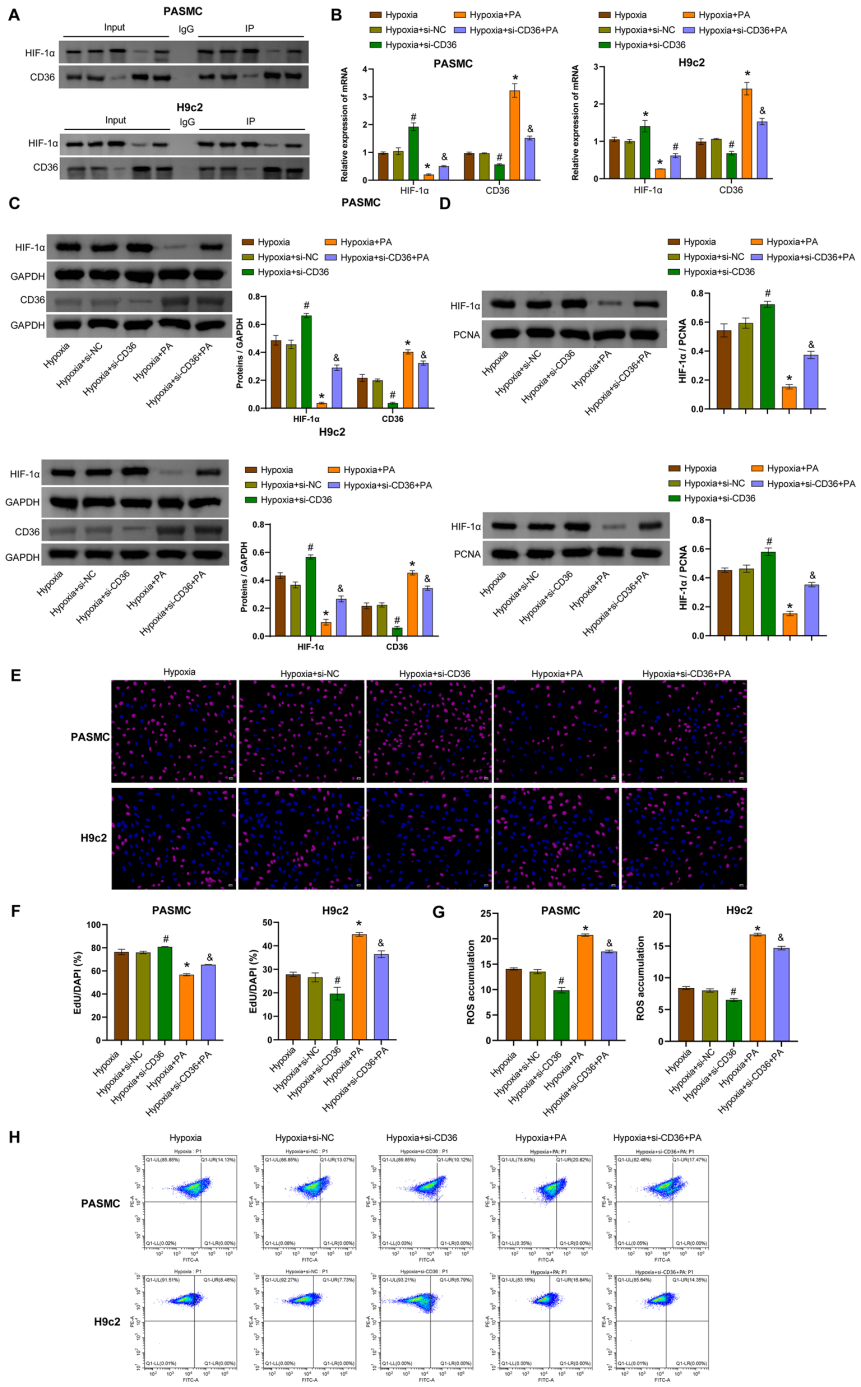

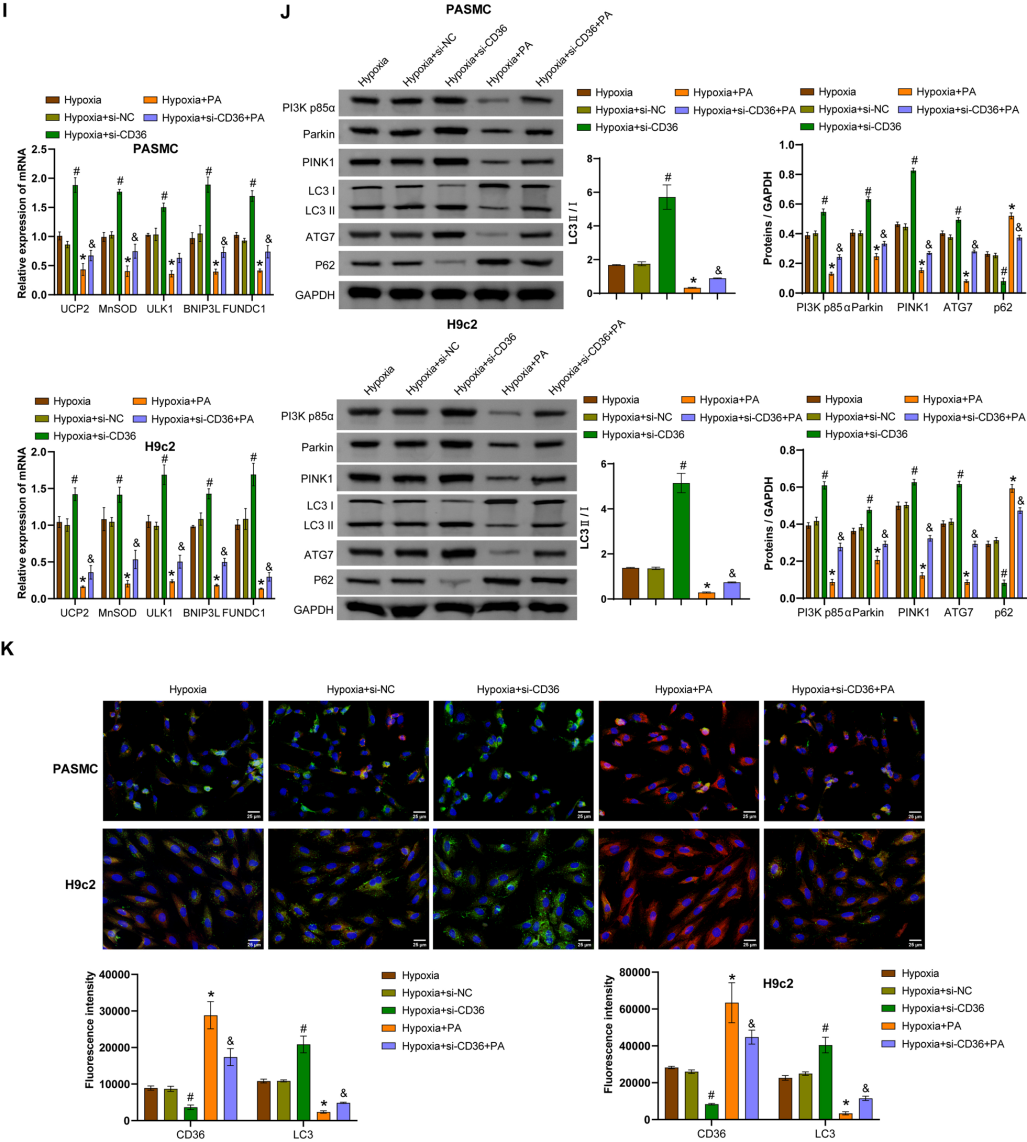
Hypoxia activated the HIF-1α/CD36 axis to regulate fatty acid uptake and mitophagy in hPASMCs and rat H9c2 cells. **A** Co-IP experiment to detect the binding of HIF-1α and CD36. **B** qRT-PCR analysis of *HIF-1α* and *CD36* mRNA levels. **C** Western blotting analysis of HIF-1α and CD36 protein levels. **D** Western blotting analysis of mitochondrial HIF-1α protein levels. **E, F** EdU staining analysis of proliferation in hPASMCs and rat H9c2 cells. Scale bar = 50 μm. **G, H** Flow cytometric analysis of ROS accumulation. **I** qRT-PCR analysis of *UCP2*, *MnSOD*, *ULK1*, *BNIP3L* and *FUNDC1* mRNA levels. **J** Western blotting analysis of PI3K p85α, Parkin, PINK1, LC3 II/I, ATG7, and p62 levels. **K** Immunofluorescence analysis of CD36 (red) and LC3 (green) expression in cells. Scale bar = 25 μm. ^*^P < 0.05 vs. Hypoxia. ^#^*P* < 0.05 vs. Hypoxia + si-NC. ^&^*P* < 0.05 vs. Hypoxia + si-CD36. n = 3. The statistical test was performed using one-way ANOVA, followed by Tukey’s post hoc test

Next, we investigated whether CD36 regulates fatty acid uptake and mitophagy in hypoxia-exposed hPASMCs and rat H9c2 cells. Co-IP analysis revealed an interaction between HIF-1α and CD36 (Fig. 3A). PA is a long-chain saturated fatty acid that can increase the uptake of exogenous PA to activate CD36 (Pan et al. 2019). Hypoxia-exposed hPASMCs and rat H9c2 cells were transfected with si-CD36 and exposed to PA treatment. Compared with the hypoxia group, PA induced CD36 expression and suppressed HIF-1α expression, whereas transfection of si-NC did not affect CD36 and HIF-1α expression. Compared with the hypoxia + si-NC group, transfection of si-CD36 inhibited CD36 expression and promoted HIF-1α expression. Compared with the hypoxia + si-CD36 group, the hypoxia + PA + si-CD36 group showed increased CD36 expression and decreased HIF-1α expression (Fig. 3B, C). Compared with the hypoxia group, PA suppressed mitochondrial HIF-1α expression, whereas transfection of si-NC did not affect HIF-1α expression. Compared with the hypoxia + si-NC group, transfection of si-CD36 promoted mitochondrial HIF-1α expression. Compared with the hypoxia + si-CD36 group, the hypoxia + PA + si-CD36 group showed decreased mitochondrial HIF-1α expression (Fig. 3C). PA treatment inhibited the proliferation of hPASMCs and promoted the proliferation of rat H9c2 cells compared with the hypoxia group. Hypoxia-challenged cells transfected with si-NC had no effect on cell proliferation. Compared with the hypoxia + si-NC group, transfection of si-CD36 promoted the proliferation of hPASMCs and attenuated the proliferation of rat H9c2 cells. Compared with the hypoxia + si-CD36 group, PA limited the proliferation of hPASMCs and enhanced the proliferation of rat H9c2 cells (Fig. 3E, F and S1B). Compared with the hypoxia group, PA treatment induced an increase in ROS levels, whereas transfection of si-NC did not affect ROS levels. Compared to the hypoxia + si-NC group, transfection of si-CD36 inhibited ROS accumulation. Compared to the hypoxia + si-CD36 group, PA-induced ROS accumulation (Fig. 3G, H). In addition, compared with the hypoxia group, PA treatment decreased the mRNA levels of *UCP2*, *MnSOD*, *ULK1*, *BNIP3L*, and *FUNDC1*, whereas transfection of si-NC did not affect the expression of these genes. Compared with the hypoxia + si-NC group, transfection with si-CD36 increased the mRNA levels of *UCP2*, *MnSOD*, *ULK1*, *BNIP3L*, and *FUNDC1*. Compared with the hypoxia + si-CD36 group, PA decreased the mRNA levels of these genes (Fig. 3I). PA inhibited the expression of PI3K p85α, Parkin, PINK, LC3 II/I, and ATG7 and increased the expression of p62 in cells exposed to hypoxia. Compared with the hypoxia + si-NC group, transfection with si-CD36 promoted the levels of PI3K p85α, Parkin, PINK, LC3 II/I, and ATG7, and restricted the expression of p62. Compared with the hypoxia + si-CD36 group, PA downregulated the expression of PI3K p85α, Parkin, PINK, LC3 II/I, and ATG7, and upregulated the expression of p62 (Fig. 3J). IF staining further confirmed that in cells challenged with hypoxia, PA induced the expression of CD36 and reduced the expression of LC3. Compared with the hypoxia + si-NC group, transfection with si-CD36 had the opposite effect, showing decreased expression of CD36 and increased expression of LC3 (Fig. 3K and S1C). Taken together, these results indicated that hypoxia activated the HIF-1α/CD36 axis to regulate fatty acid uptake, autophagy-related markers, and Parkin/PINK1.

### PI3K p85α/HIF-1α regulates proliferation and mitophagy in hPASMCs and rat H9c2 cells exposed to hypoxia via the Parkin/PINK1 pathway

**Fig. 4:**
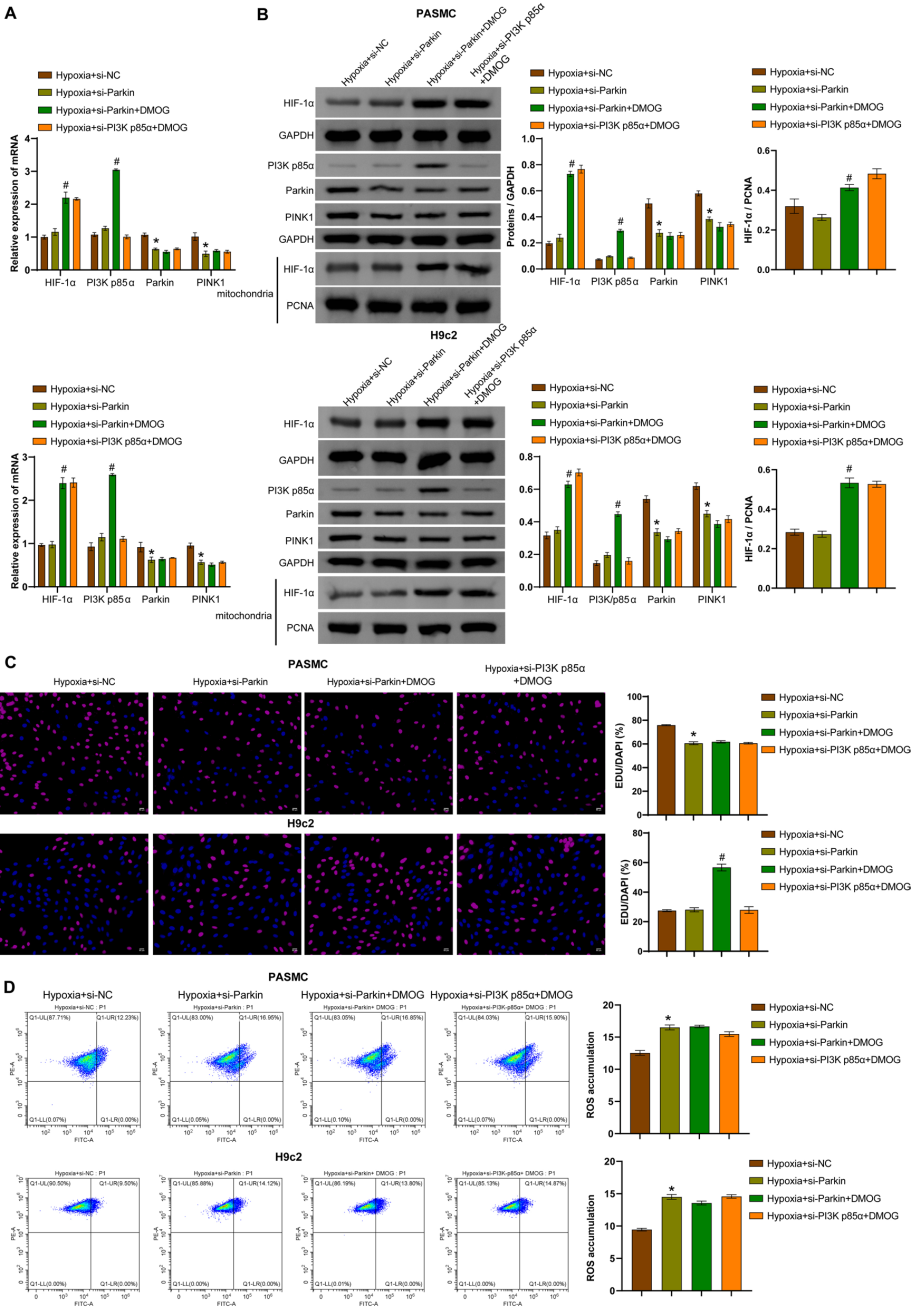

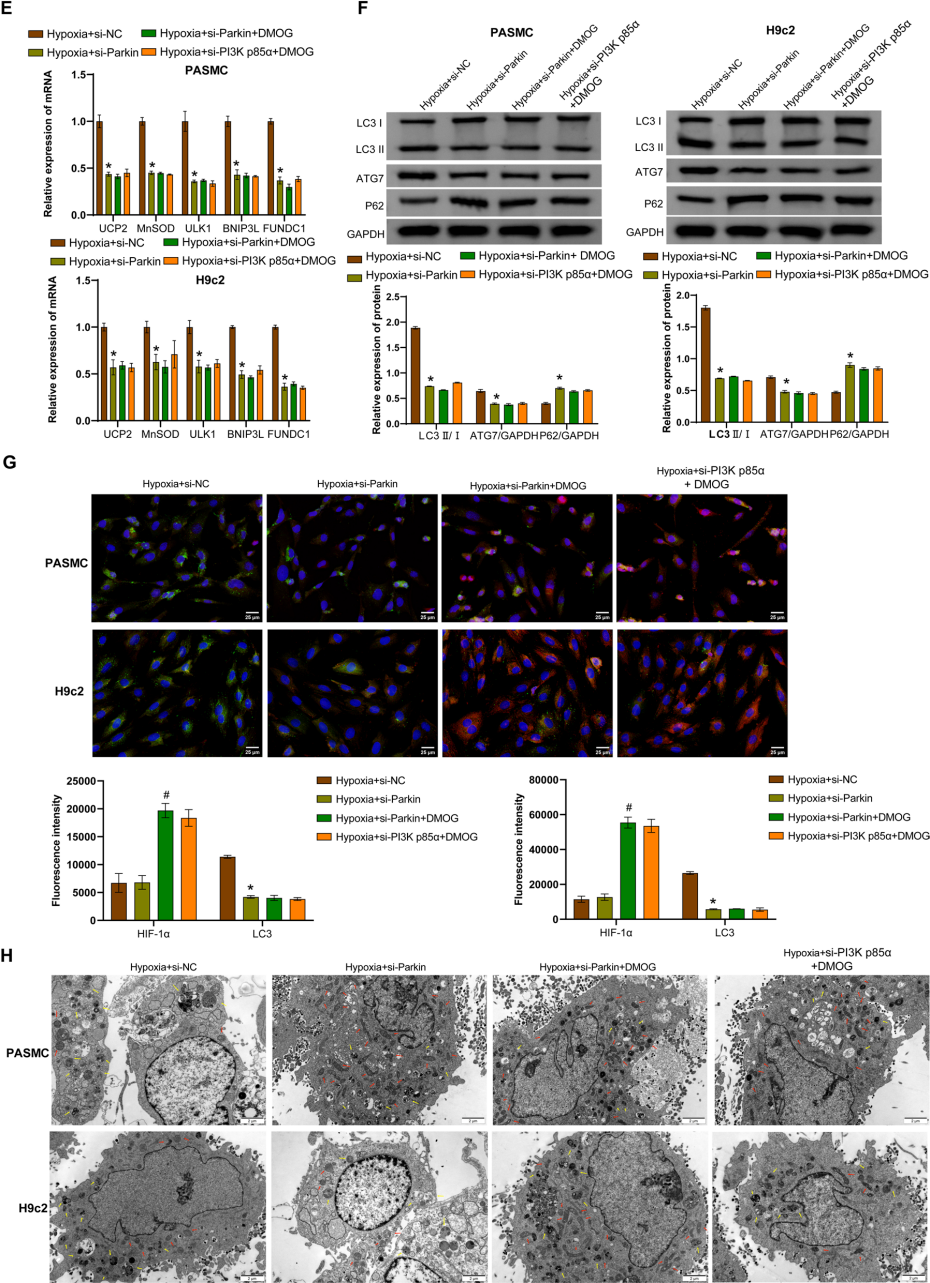
Hypoxia activated the PI3K p85α/HIF-1αaxis to regulate proliferation and mitophagy in hPASMCs and rat H9c2 cells through the Parkin/PINK1 pathway. A qRT-PCR analysis of *HIF-1α*, *PI3K p85α*, *Parkin* and *PINK1* mRNA levels. **B** Western blotting analysis of HIF-1α, PI3K p85α, Parkin, PINK1, and mitochondrial HIF-1α protein levels. **C** EdU staining assessment of proliferation in hPASMCs and rat H9c2 cells. Scale bar = 50 μm. **D** Flow cytometry analysis of ROS accumulation. **E** qRT-PCR analysis of *UCP2*, *MnSOD*, *ULK1*, *BNIP3L* and *FUNDC1* mRNA levels. **F** Western blotting analysis of LC3 II/I, ATG7, and p62 levels. **G** Immunofluorescence analysis of HIF-1α (red) and LC3 (green) expression in cells. Scale bar = 25 μm. **H** Transmission electron microscopy observation of mitochondrial structure and autophagosome formation. Autophagosomes are indicated by yellow arrows and relatively normal mitochondria are indicated by red arrows. Scale bar = 2 μm. ^*^P < 0.05 vs. Hypoxia + si-NC. ^#^*P* < 0.05 vs. Hypoxia + si-Parkin. n = 3. The statistical test was performed using one-way ANOVA, followed by Tukey’s post hoc test

Hypoxia-exposed hPASMCs and rat H9c2 cells were transfected with si-Parkin or si-PI3K p85α, followed by treatment with the HIF-1α activator DMOG. Compared with the hypoxia + si-NC group, transfection with si-Parkin restricted the expression of Parkin and PINK1, but did not affect the expression of HIF-1α and PI3K p85α. Compared with the hypoxia + si-Parkin group, DMOG increased the expression of HIF-1α and PI3K p85α, but had no effect on the expression of Parkin and PINK1 (Fig. 4A, B). Moreover, compared with the hypoxia + si-Parkin group, DMOG increased mitochondrial HIF-1α expression (Fig. 4B). Transfection with si-Parkin reduced hPASMC proliferation, but did not affect proliferation of rat H9c2 cells compared to the hypoxia + si-NC group. DMOG increased proliferation of rat H9c2 cells, but had no effect on proliferation of hPASMCs (Fig. 4C and S1D). Transfection with si-Parkin promoted ROS accumulation compared to the hypoxia + si-NC group, whereas further treatment with DMOG did not affect ROS levels (Fig. 4D). Compared with the Hypoxia + si-NC group, transfection with si-Parkin resulted in decreased levels of *UCP2*, *MnSOD*, *ULK1*, *BNIP3L*, and *FUNDC1*, while further treatment with DMOG did not affect the levels of these genes (Fig. 4E). Transfection with si-Parkin also decreased LC3 II/I ratio and ATG7 expression and increased p62 expression compared to the hypoxia + si-NC group, while further treatment with DMOG did not affect the expression of these proteins (Fig. 4F). IF staining showed that transfection with si-Parkin did not affect the fluorescence intensity of HIF-1α, but decreased the fluorescence intensity of LC3 compared with the Hypoxia + si-NC group. Compared with the hypoxia + si-Parkin group, DMOG increased the fluorescence intensity of HIF-1α but did not affect the fluorescence intensity of LC3 (Fig. 4G and S1E). TEM observation revealed that transfection with si-Parkin reduced the number of autophagosomes and damaged mitochondria in hypoxic cells, while further treatment with DMOG did not cause significant changes in the number (Fig. 4H). In addition, the hypoxia + si-Parkin + DMOG group and the hypoxia + si-PI3K p85α + DMOG group showed similar effects (Fig. 4). Collectively, these results indicated that PI3K p85α/HIF-1α stimulated the Parkin/PINK1 pathway to induce mitophagy in hPASMCs and rat H9c2 cells and enhance hPASMC proliferation.

### HIF-1α regulates the pathological symptoms in MCT-induced PAH-like rats by modulating the CD36 and Parkin/PINK1 signaling pathways

**Fig. 5:**
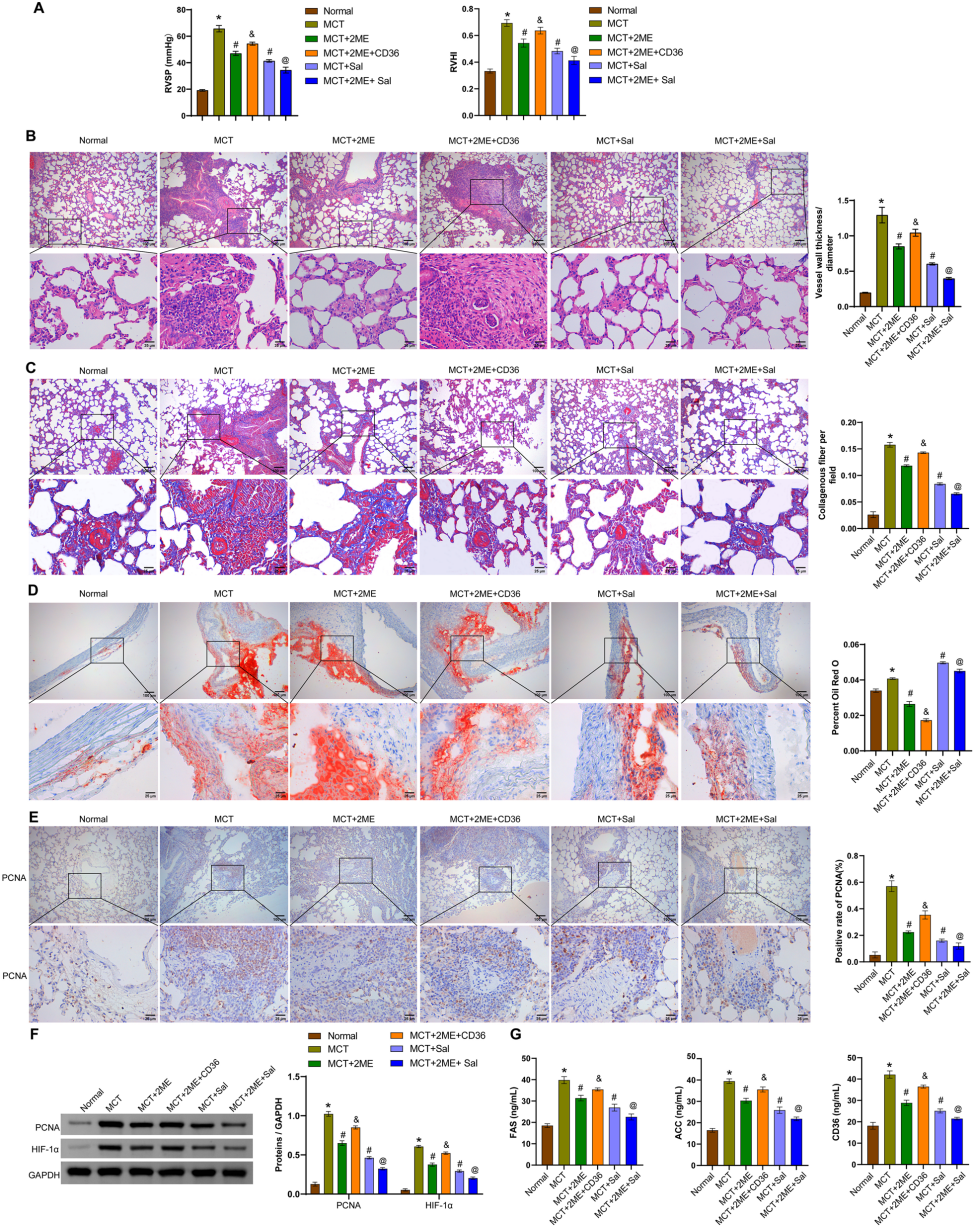
HIF-1α regulated right ventricular function and pathological manifestations in MCT-induced PAH-like rats through modulation of CD36 and Parkin/PINK1 signaling pathways. **A** Measurement of RVSP and RVHI. **B** Representative HE staining of lung tissues and quantitative analysis of vessel wall thickness/diameter ratio. Scale bar = 100 μm. **C** Representative Masson’s trichrome staining of lung tissues and quantitative analysis of collagen deposition. Scale bar = 100 μm. **D** Representative Oil Red O staining of lung tissues and quantitative analysis of positive staining. Scale bar = 100 μm. **E** Representative immunohistochemistry images and statistical analysis of PCNA-positive staining in lung tissues. **F** Western blotting analysis of PCNA and HIF-1α protein levels. **G** Serum levels of FAS, ACC, and CD36. ^*^P < 0.05 vs. Normal. ^#^*P* < 0.05 vs. PAH. ^&^*P* < 0.05 vs. PAH + 2ME. ^@^*P* < 0.05 vs. PAH + Sal. n = 6. The statistical test was performed using one-way ANOVA, followed by Tukey’s post hoc test

To investigate whether HIF-1α could treat right ventricular dysfunction in the PAH-like rat model, RVSP and RVHI was measured. Compared with the normal group, MCT increased RVSP and RVHI, whereas injection of the HIF-1α antagonist 2ME or the Parkin/PINK1 pathway activator Sal decreased RVSP and RVHI. Supplementation with recombinant CD36 increased RVSP and RVHI compared to the PAH + 2ME group. Compared to the PAH + Sal group, 2ME further reduced RVSP and RVHI (Fig. 5A). Histologic studies were then performed to demonstrate the effect of HIF-1α on pulmonary vascular remodeling during the development of PAH. Using tissue stainings, MCT-induced PAH-like rats exhibited thickened pulmonary artery walls, narrowed lumens, perivascular collagen deposition, and lipid deposition. Intervention with 2ME or Sal reduced pulmonary artery wall thickening, collagen deposition, and lipid deposition in PAH-like rats. Supplementation with recombinant CD36 reversed the protective effect of 2ME compared to the PAH + 2ME group. Compared with the PAH + Sal group, 2ME enhanced the protective effect of Sal (Fig. 5B–D). In addition, MCT-induced rats showed increased expression of PCNA and HIF-1α in lung tissue. Supplementation with 2ME or Sal reversed the expression of these three proteins. Compared with the PAH + 2ME group, supplementation with recombinant CD36 increased the expression of PCNA and HIF-1α. Compared with the PAH + Sal group, 2ME further downregulated the expression of PCNA and HIF-1α (Fig. 5E, F). The levels of fatty acid synthesis/uptake-related indicators FAS, ACC, and CD36 were elevated in the peripheral blood of MCT-induced PAH-like rats, and supplementation with 2ME or Sal reversed these levels. Compared with the PAH + 2ME group, supplementation with recombinant CD36 increased the levels of FAS, ACC, and CD36. Compared with the PAH + Sal group, 2ME further decreased the levels of FAS, ACC, and CD36 (Fig. 5G). Taken together, these results suggested that the HIF-1α antagonist alleviated the pathological symptoms of MCT-induced PAH-like rats by regulating fatty acid uptake via CD36 and the Parkin/PINK1 signaling pathway.

### HIF-1α regulates fatty acid metabolism and mitophagy in MCT-induced PAH-like rats by modulating the CD36 and Parkin/PINK1 signaling pathways

**Fig. 6:**
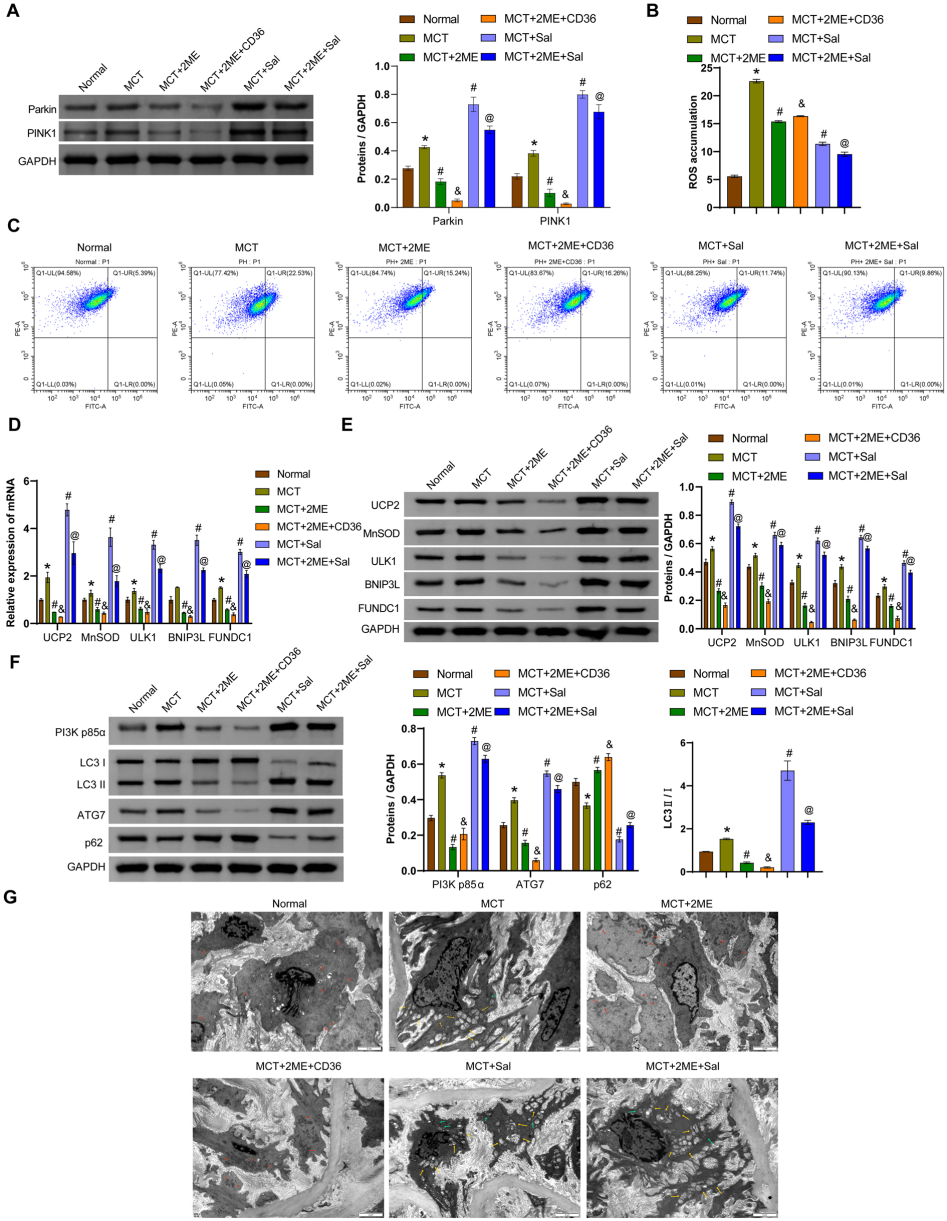
HIF-1α regulated fatty acid uptake and mitophagy in MCT-induced PAH-like rats through modulation of CD36 and Parkin/PINK1 pathways. **A** Western blotting analysis of Parkin and PINK1 in lung tissues. **B**, **C** Detection of ROS accumulation in lung tissue by flow cytometry. **D** qRT-PCR measurement of *UCP2*, *MnSOD*, *ULK1*, *BNIP3L* and *FUNDC1* mRNA levels in lung tissues. **E** Western blotting analysis of UCP2, MnSOD, ULK1, BNIP3L, and FUNDC1 levels in lung tissues. **F** Western blotting analysis of PI3K p85α, LC3 II/I, ATG7, and p62 levels in lung tissues. **G** Transmission electron microscopy observation of mitochondrial structure and autophagosome formation in lung tissues. Yellow arrows indicate autophagosomes, green indicate damaged mitochondria and red arrows indicate relatively normal mitochondria. Scale bar = 2 μm. ^*^P < 0.05 vs. Normal. ^#^*P* < 0.05 vs. PAH. ^&^*P* < 0.05 vs. PAH + 2ME. ^@^*P* < 0.05 vs. PAH + Sal. n = 6. The statistical test was performed using one-way ANOVA, followed by Tukey’s post hoc test

Compared with the normal rats, MCT increased the expression of Parkin and PINK1. Injection of 2ME decreased the levels of Parkin and PINK1 in PAH-like rats, while injection of Sal increased the expression of these two proteins. Supplemental recombinant CD36 further decreased the levels of Parkin and PINK1 compared to the PAH + 2ME group (Fig. 6A and S2). ROS accumulation was observed in lung tissues of PAH-like rats, 2ME and Sal injections both reduced ROS accumulation. CD36 intervention increased ROS accumulation compared with the PAH + 2ME group. Compared with the PAH + Sal group, 2ME reduced ROS accumulation (Fig. 6B, C). 2ME decreased the expression of UCP2, MnSOD, ULK1, BNIP3L and FUNDC1 in the pulmonary artery tissues of PAH-like rats, while CD36 supplementation further reduced the levels of these factors. Sal intervention increased the levels of UCP2, MnSOD, ULK1, BNIP3L, and FUNDC1 in the PAH-like rats, and supplementation with 2ME reversed these levels (Fig. 6D, E). In addition, upregulation of LC3 II/I, PI3K p85α, and ATG7 and downregulation of p62 were observed in lung tissues of PAH-like rats. 2ME intervention reversed the expression of these proteins in PAH-like rats, and CD36 intervention further enhanced the effect of 2ME. Sal intervention upregulated the ratio of LC3 II/I, expression of PI3K p85α and ATG7, and downregulated p62 expression in PAH-like rats, and further application of 2ME reversed the expression of these proteins (Fig. 6F). Increased number of mitochondrial damage and autophagosomes were also observed in PAH-like rats. 2ME intervention reduced this number in PAH-like rats, and the addition of CD36 exerted further protective effects. Sal intervention increased the number of damaged mitochondria and autophagosomes in PAH-like rats, while 2ME supplementation helped to alleviate this phenomenon (Fig. 6G). These results suggested that HIF-1α antagonists regulated fatty acid uptake and mitophagy in MCT-induced PAH-like rats by modulating the CD36 and Parkin/PINK1 signaling pathways.

## Discussion

HIF-1α regulates a number of transcriptional responses in response to hypoxia. As a key transcriptional regulator, activated HIF-1α binds to hypoxia response elements (HRE) and induces transcription of genes including those involved in angiogenesis, cell survival and proliferation, and metabolism (Tirpe et al. 2019). Hypoxia-induced HIF-1α promotes PASMC proliferation and pulmonary vascular remodeling during PAH (Wilkins et al. 2015). Despite these findings, the regulatory network mediated by HIF-1α and the development of effective therapeutic strategies for PAH remains largely unclear. In this study, we investigated HIF-1α-mediated fatty acid uptake and mitophagy under hypoxic conditions involving activation of the CD36 and Parkin/PINK1 signaling pathways. Activation of the HIF-1α-CD36 and HIF-1α-Parkin/PINK1 axis triggered pulmonary vascular remodeling and accelerated the progression of PAH.

Mitochondria play a critical role in maintaining cellular homeostasis and are implicated in the pathogenesis of PAH. PAH is characterized as a syndrome caused in part by acquired mitochondrial dysfunction (Breault et al. 2023). Mitochondrial dysfunction leads to excessive proliferation of PASMCs, accumulation of mitochondrial ROS, and activation of inflammatory responses (Culley and Chan 2018). PASMCs and myocardial cells respond to stress by inducing mitophagy in an attempt to eliminate damaged mitochondria and restore mitochondrial function (Alves-Silva et al. 2022; Liu et al. 2023). In this study, the upregulation of HIF-1α in the serum of PAH patients was positively correlated with the serum levels of Parkin/PINK1, CD36, FAS, and ACC. Mitochondrial dysfunction may be systemic (Gezen-Ak et al. 2020). Previous studies have reported mitochondrial oxidative stress and impaired mitophagy in the peripheral blood mononuclear cell (PBMC) of obese subjects with metabolic abnormalities (Bhansali et al. 2017a). TEM observations reveal altered mitochondrial morphology in PBMCs of patients with type 2 diabetes (Bhansali et al. 2017b). In addition, Parkin/PINK1 levels are altered in PBMCs of patients with type 2 diabetes, and mitophagy is reversed in PBMCs of patients treated with metformin (Marañón et al. 2022). The expression profile of PBMCs of patients with PAH has been shown to predict disease risk and participate in disease mechanisms (Romanoski et al. 2020). Based on transcriptome data, gene sets corresponding to hypoxia were differentially expressed in PBMCs of patients with PAH (Zhu et al. 2021). Thus, PBMC data may suggest candidate factors associated with the development of PAH, including HIF-1α, Parkin/PINK1, and CD36. More clinical samples need to be collected to confirm the crosstalk between HIF-1α, mitophagy, and fatty acid metabolism.

The present study indicated that HIF-1α knockdown reversed the activation of the Parkin/PINK1 pathway in hypoxia-induced hPASMCs and rat H9c2 cells. Parkin knockdown resulted in decreased proliferation and reduced mitophagy in hPASMCs under hypoxic conditions. Additional administration of the HIF-1α activator DMOG did not alter the consequences of Parkin knockdown. These findings indicated that hypoxia-induced activation of HIF-1α promoted mitophagy through the Parkin/PINK1 pathway, thereby reducing hPASMC proliferation and increasing rat H9c2 cell proliferation. The Parkin/PINK1-dependent classical ubiquitination pathway is a key mechanism driving mitophagy. In addition to the ubiquitin-dependent pathway, adapter proteins located on the outer mitochondrial membrane, such as BNIP3L and FUNDC1, can directly interact with LC3 without the involvement of ubiquitination, thereby initiating mitophagy (Su et al. 2022). The ubiquitin-independent pathway is closely related to the Parkin/PINK1 pathway. As an autophagy initiator, ULK1 phosphorylates several key autophagy-related factors to accelerate mitophagy (Poole et al. 2021). UCP2 and MnSOD play important roles in regulating mitochondrial oxidative stress and function (Tian et al. 2018; Greenberger et al. 2021). Here, we observed that HIF-1α knockdown or Parkin knockdown reduced hypoxia-induced upregulation of LC3, ATG7, UCP2, MnSOD, ULK1, BNIP3L, and FUNDC1 levels. These results further suggested that HIF-1α regulated mitochondrial oxidative stress and mitophagy in hPASMCs and rat H9c2 cells through the Parkin/PINK1 pathway.

The stability of HIF-1α under hypoxic conditions depends on the PI3K/protein kinase B (Akt)/mTOR signaling pathway (Xie et al. 2019). PI3Ks are lipid kinases, and p85α is an important regulatory subunit of PI3K (Chen et al. 2022a). PI3K can be phosphorylated and activated by p85α, which affects the activity of PI3K protein. In this study, knockdown of PI3K p85α downregulated the expression of HIF-1α in hypoxia-exposed hPASMCs and rat H9c2 cells. Knockdown of PI3K p85α also reduced hypoxia-induced proliferation in hPASMCs and increased proliferation in rat H9c2 cells exposed to hypoxia. In addition, knockdown of PI3K p85α attenuated hypoxia-induced mitochondrial oxidative damage and reduced mitophagy. The addition of DMOG did not alter the effects of PI3K p85α knockdown. These results suggested that the PI3K pathway could act as a regulatory signal of HIF-1α and mediate HIF-1α expression.

In this study, we observed an increase in key limiting enzymes of fatty acid synthesis (FAS and ACC) and fatty acid transporter protein (CD36) in the serum of PAH patients and MCT-induced PAH-like rats, suggesting fatty acid metabolism dysregulation during PAH. The present study focused on the role of CD36. CD36, located on the cell membrane, is responsible for transporting fatty acids taken up from the extracellular environment and can also respond to extracellular signals to transduce signals to various downstream effectors (Chen et al. 2022b; Samovski et al. 2023). In PAH, increased CD36-mediated fatty acid uptake and imbalanced mitochondrial glucose and fatty acid oxidation cause right ventricular dysfunction (Talati and Hemnes 2015). Here, hypoxia increased CD36 expression, whereas knockdown of HIF-1α reversed this trend. In hypoxia-exposed hPASMCs and rat H9c2 cells, CD36 knockdown enhanced the expression of HIF-1α, PI3K p85α, Parkin, and PINK1, increased hPASMC proliferation and decreased H9c2 cell proliferation. MCT induced an increase in fatty acid uptake and lipid accumulation, while the HIF-1α antagonist 2ME reduced this phenomenon in MCT-induced PAH-like rats. Interestingly, we observed that supplementation of CD36 increased HIF-1α expression in MCT-induced PAH-like rats after 2ME injection, exacerbating pulmonary artery injury and lipid accumulation. These results suggested that HIF-1α may signal through increased CD36 expression, and in turn, increased CD36 activity helped increase the ability of cells to take up fatty acids, resulting in high expression of HIF-1α.

Fatty acid metabolism, particularly β-oxidation, is closely associated with mitochondria. High expression of CD36 leads to high levels of fatty acid oxidation and uptake of free fatty acids, providing energy for cell generation (Mistry et al. 2021). In hepatocytes and renal tubular epithelial cells, CD36-mediated fatty acid transport is critical for fatty acid oxidation and affects mitochondrial function (Niu et al. 2023; Zeng et al. 2022). In this study, CD36 knockdown reduced ROS accumulation and exacerbated mitophagy in hypoxia-exposed hPASMCs and rat H9c2 cells. Exogenous saturated fatty acids, such as PA, lead to intracellular lipid accumulation, ROS production, and mitochondrial dysfunction (Alnahdi et al. 2019). PA treatment decreases Parkin expression in primary hepatocytes and inhibits mitophagy (Zhou et al. 2019). Here, the addition of PA alone resulted in significantly different results than CD36 knockdown. The addition of PA inhibited CD36 knockdown induced Parkin/PINK1-dependent mitophagy. In MCT-induced PAH-like rats after 2ME injection, CD36 supplementation further inhibited mitophagy. Furthermore, compared with untreated PAH-like rats, administration of the Parkin/PINK1 pathway activator Sal alone promoted mitophagy, whereas further addition of 2ME reversed this trend. We speculated that the contradictory role of CD36 may be due to the fact that in MCT-induced PAH-like rats, 2ME led to decreased HIF-1α activity, resulting in reduced CD36-mediated fatty acid uptake and mitophagy inhibition. However, further exogenous supplementation of CD36 increased CD36 activity, increased fatty acid uptake, causing mitochondrial fatty acid oxidation overload and impairing mitophagy. Additional experimental evidence is needed to understand the regulatory role of the HIF-1α-CD36 axis in fatty acid metabolism and mitochondrial function in PAH. All of the above findings require further validation in a more comprehensive clinical sample. Our study needs to be confirmed in more models, such as the hypoxia-sugen model. The mechanism of HIF-1α-mediated mitophagy in PAH requires further investigation.

## Conclusion

In conclusion, this study demonstrated that activated HIF-1α promoted the expression of CD36 and Parkin/PINK1-dependent mitophagy in PASMCs and cardiomyocytes challenged with hypoxia. The activity of HIF-1α was regulated by PI3K p85α and affected cell proliferation and mitochondrial function. Furthermore, in MCT-induced PAH-like rats, HIF-1α antagonists helped alleviate pulmonary vascular remodeling and lipid accumulation, but supplementation of recombinant CD36 affected the therapeutic effect. These findings may provide new insights for the development of therapeutic strategies targeting PAH.

## Supplementary Information

Additional file 1: Figure S1. Representative fluorescence images. (A) Representative fluorescence images of DAPI (blue) and EdU (red) labeling in 2D. (B) Representative fluorescence images of DAPI (blue) and EdU (red) labeling in 3E. (C) Representative fluorescence images of DAPI (blue), CD36 (red), and LC3 (green) labeling in 3K. (D) Representative fluorescence images of DAPI (blue) and EdU (red) labeling in 4C. (E) Representative fluorescence images of DAPI (blue), HIF-1α (red), and LC3 (green) labeling in 4G.

Additional file 2: Figure S2. Representative immunohistochemistry images and statistical analysis of Parkin and PINK1 in lung tissue. *P < 0.05 vs. Normal. #P < 0.05 vs. PAH. &P < 0.05 vs. PAH+2ME. @P < 0.05 vs. PAH+Sal. n = 6. The statistical test was performed using one-way ANOVA, followed by Tukey’s post hoc test.

Additional file 3.

Additional file 4.

Additional file 5.

## Data Availability

Data will be made available on request.

## References

[CR1] Alnahdi A, John A, Raza H. Augmentation of glucotoxicity, oxidative stress, apoptosis and mitochondrial dysfunction in HepG2 cells by palmitic acid. Nutrients. 2019. 10.3390/nu11091979. 31443411 10.3390/nu11091979 PMC6770774

[CR2] Alves-Silva JM, Zuzarte M, Marques C, Viana S, Preguiça I, Baptista R, Ferreira C, Cavaleiro C, Domingues N, Sardão VA, Oliveira PJ, Reis F, Salgueiro L, Girão H. 1,8-Cineole ameliorates right ventricle dysfunction associated with pulmonary arterial hypertension by restoring connexin43 and mitochondrial homeostasis. Pharmacol Res. 2022. 10.1016/j.phrs.2022.106151. 35247601 10.1016/j.phrs.2022.106151

[CR3] Aoki T, Kinoshita J, Munesue S, Hamabe-Horiike T, Yamaguchi T, Nakamura Y, Okamoto K, Moriyama H, Nakamura K, Harada S, Yamamoto Y, Inaki N, Fushida S. Hypoxia-induced CD36 Expression in gastric cancer cells promotes peritoneal metastasis via fatty acid uptake. Ann Surg Oncol. 2022;30:3125–36. 10.1245/s10434-022-12465-5. 36042102 10.1245/s10434-022-12465-5 PMC10085939

[CR4] Arafah MM, Al-Qattan MM, Abdulmaged-Ahmed DA, Al-Nafesah GA, Jadu NY, Bagayawa RS, Shier MK, Marzouk A, Almalki HS. The use of antifibrotic recombinant nAG protein in a rat liver fibrosis model. Biomed Res Int. 2019;2019:9846919. 10.1155/2019/9846919. 31275996 10.1155/2019/9846919 PMC6582902

[CR5] Azhar AS, Zaher ZF, Ashour OM, Abdel-Naim AB. 2-Methoxyestradiol ameliorates metabolic syndrome-induced hypertension and catechol-O-methyltransferase inhibited expression and activity in rats. Eur J Pharmacol. 2020;882: 173278. 10.1016/j.ejphar.2020.173278. 32544503 10.1016/j.ejphar.2020.173278

[CR6] Bai Y-T, Xiao F-J, Wang H, Ge R-L, Wang L-S. Hypoxia protects H9c2 cells against Ferroptosis through SENP1-mediated protein DeSUMOylation. Int J Med Sci. 2021;18:1618–27. 10.7150/ijms.50804. 33746578 10.7150/ijms.50804 PMC7976564

[CR7] Beshay S, Sahay S, Humbert M. Evaluation and management of pulmonary arterial hypertension. Respir Med. 2020;171: 106099. 10.1016/j.rmed.2020.106099. 32829182 10.1016/j.rmed.2020.106099

[CR8] Bhansali S, Bhansali A, Dhawan V. Favourable metabolic profile sustains mitophagy and prevents metabolic abnormalities in metabolically healthy obese individuals. Diabetol Metab Syndr. 2017a;9:99. 10.1186/s13098-017-0298-x. 29255491 10.1186/s13098-017-0298-x PMC5728047

[CR9] Bhansali S, Bhansali A, Walia R, Saikia UN, Dhawan V. Alterations in mitochondrial oxidative stress and mitophagy in subjects with prediabetes and type 2 diabetes mellitus. Front Endocrinol (Lausanne). 2017b;8:347. 10.3389/fendo.2017.00347. 29326655 10.3389/fendo.2017.00347 PMC5737033

[CR10] Breault NM, Wu D, Dasgupta A, Chen K-H, Archer SL. Acquired disorders of mitochondrial metabolism and dynamics in pulmonary arterial hypertension. Front Cell Dev Biol. 2023. 10.3389/fcell.2023.1105565. 36819102 10.3389/fcell.2023.1105565 PMC9933518

[CR11] Chen LF, Xu WB, Xiong S, Cai JX, Zhang JJ, Li YL, Li MM, Zhang H, Liu Z. PIK-24 inhibits RSV-induced syncytium formation via direct interaction with the p85α subunit of PI3K. J Virol. 2022a;96:e0145322. 36416586 10.1128/jvi.01453-22 PMC9749462

[CR12] Chen Y, Zhang J, Cui W, Silverstein RL. CD36, a signaling receptor and fatty acid transporter that regulates immune cell metabolism and fate. J Exp Med. 2022b. 10.1084/jem.20211314. 35438721 10.1084/jem.20211314 PMC9022290

[CR13] Chen R, Ahmed MA, Forsyth NR. Dimethyloxalylglycine (DMOG), a hypoxia mimetic agent, does not replicate a rat pheochromocytoma (PC12) cell biological response to reduced oxygen culture. Biomolecules. 2022c;12:541. 35454130 10.3390/biom12040541 PMC9027160

[CR14] Chen Y-X, Deng Z-H, Xue G, Qiang D, Juan Y, Chen G-H, Li J-G, Zhao Y-M, Zhang H-T, Zhang G-X, Qian J-X. Exosomes derived from mesenchymal stromal cells exert a therapeutic effect on hypoxia-induced pulmonary hypertension by modulating the YAP1/SPP1 signaling pathway. Biomed Pharmacother. 2023. 10.1016/j.biopha.2023.115816. 37918254 10.1016/j.biopha.2023.115816

[CR15] Chen L, Lv Y, Wu H, Wang Y, Xu Z, Liu G, He Y, Li X, Liu J, Feng Y, Bai Y, Xie W, Zhou Q, Wu Q. Gastrodin exerts perioperative myocardial protection by improving mitophagy through the PINK1/Parkin pathway to reduce myocardial ischemia-reperfusion injury. Phytomedicine. 2024;133: 155900. 10.1016/j.phymed.2024.155900. 39094441 10.1016/j.phymed.2024.155900

[CR16] Culley MK, Chan SY. Mitochondrial metabolism in pulmonary hypertension: beyond mountains there are mountains. J Clin Investig. 2018;128:3704–15. 10.1172/jci120847. 30080181 10.1172/JCI120847 PMC6118596

[CR17] de Marañón AM, Díaz-Pozo P, Canet F, Díaz-Morales N, Abad-Jiménez Z, López-Domènech S, Vezza T, Apostolova N, Morillas C, Rocha M, Víctor VM. Metformin modulates mitochondrial function and mitophagy in peripheral blood mononuclear cells from type 2 diabetic patients. Redox Biol. 2022;53: 102342. 10.1016/j.redox.2022.102342. 35605453 10.1016/j.redox.2022.102342 PMC9124713

[CR18] Feng M, Zhou Q, Xie H, Liu C, Zheng M, Zhang S, Zhou S, Zhao J. Role of CD36 in central nervous system diseases. Neural Regen Res. 2024;19:512–8. 10.4103/1673-5374.380821. 37721278 10.4103/1673-5374.380821 PMC10581564

[CR19] Gezen-Ak D, Alaylıoğlu M, Genç G, Şengül B, Keskin E, Sordu P, Güleç ZEK, Apaydın H, Bayram-Gürel Ç, Ulutin T, Yılmazer S, Ertan S, Dursun E. Altered transcriptional profile of mitochondrial DNA-encoded OXPHOS subunits, mitochondria quality control genes, and intracellular ATP levels in blood samples of patients with Parkinson’s disease. J Alzheimers Dis. 2020;74:287–307. 10.3233/jad-191164. 32007957 10.3233/JAD-191164

[CR20] Greenberger JS, Mukherjee A, Epperly MW. Gene therapy for systemic or organ specific delivery of manganese superoxide dismutase. Antioxidants. 2021. 10.3390/antiox10071057. 34208819 10.3390/antiox10071057 PMC8300724

[CR21] Gu C, Li L, Huang Y, Qian D, Liu W, Zhang C, Luo Y, Zhou Z, Kong F, Zhao X, Liu H, Gao P, Chen J, Yin G. Salidroside ameliorates mitochondria-dependent neuronal apoptosis after spinal cord ischemia-reperfusion injury partially through inhibiting oxidative stress and promoting mitophagy. Oxid Med Cell Longev. 2020;2020:3549704. 10.1155/2020/3549704. 32774670 10.1155/2020/3549704 PMC7396093

[CR22] He Z, Dai L, Zuo Y, Chen Y, Wang H, Zeng H. Hotspots and frontiers in pulmonary arterial hypertension research: a bibliometric and visualization analysis from 2011 to 2020. Bioengineered. 2022a;13:14667–80. 10.1080/21655979.2022.2100064. 35880647 10.1080/21655979.2022.2100064 PMC9342150

[CR23] He J, Qin Z, Chen X, He W, Li D, Zhang L, Le Y, Xiong Q, Zhang B, Wang H. HIF-1α ameliorates diabetic neuropathic pain via Parkin-mediated mitophagy in a mouse model. Biomed Res Int. 2022b;2022:5274375. 10.1155/2022/5274375. 36017378 10.1155/2022/5274375 PMC9398773

[CR24] Hong L, Zha Y, Wang C, Qiao S, An J. Folic acid alleviates high glucose and fat-induced pyroptosis via inhibition of the hippo signal pathway on H9C2 cells. Front Mol Biosci. 2021;8: 698698. 10.3389/fmolb.2021.698698. 34692767 10.3389/fmolb.2021.698698 PMC8529044

[CR25] Jin H, Jiao Y, Guo L, Ma Y, Zhao R, Li X, Shen L, Zhou Z, Kim SC, Liu J. Astragaloside IV blocks monocrotaline-induced pulmonary arterial hypertension by improving inflammation and pulmonary artery remodeling. Int J Mol Med. 2021;47:595–606. 33416126 10.3892/ijmm.2020.4813 PMC7797426

[CR26] Lau EM, Giannoulatou E, Celermajer DS, Humbert M. Epidemiology and treatment of pulmonary arterial hypertension. Nat Rev Cardiol. 2017;14:603–14. 28593996 10.1038/nrcardio.2017.84

[CR27] Li R, Chen J. Salidroside protects dopaminergic neurons by enhancing PINK1/Parkin-mediated mitophagy. Oxid Med Cell Longev. 2019;2019:9341018. 10.1155/2019/9341018. 31583052 10.1155/2019/9341018 PMC6754964

[CR28] Li J, Li M, Ge Y, Chen J, Ma J, Wang C, Sun M, Wang L, Yao S, Yao C. β-amyloid protein induces mitophagy-dependent ferroptosis through the CD36/PINK/PARKIN pathway leading to blood–brain barrier destruction in Alzheimer’s disease. Cell Biosci. 2022. 10.1186/s13578-022-00807-5. 35619150 10.1186/s13578-022-00807-5 PMC9134700

[CR29] Liu R, Xu C, Zhang W, Cao Y, Ye J, Li B, Jia S, Weng L, Liu Y, Liu L, Zheng M. FUNDC1-mediated mitophagy and HIF1alpha activation drives pulmonary hypertension during hypoxia. Cell Death Dis. 2022;13:634. 10.1038/s41419-022-05091-2. 35864106 10.1038/s41419-022-05091-2 PMC9304375

[CR30] Liu M, He H, Fan F, Qiu L, Zheng F, Guan Y, Yang G, Chen L. Maresin-1 protects against pulmonary arterial hypertension by improving mitochondrial homeostasis through ALXR/HSP90α axis. J Mol Cell Cardiol. 2023;181:15–30. 10.1016/j.yjmcc.2023.05.005. 37244057 10.1016/j.yjmcc.2023.05.005

[CR31] Lu Y, Li Z, Zhang S, Zhang T, Liu Y, Zhang L. Cellular mitophagy: mechanism, roles in diseases and small molecule pharmacological regulation. Theranostics. 2023;13:736–66. 10.7150/thno.79876. 36632220 10.7150/thno.79876 PMC9830443

[CR32] Luo Y, Teng X, Zhang L, Chen J, Liu Z, Chen X, Zhao S, Yang S, Feng J, Yan X. CD146-HIF-1alpha hypoxic reprogramming drives vascular remodeling and pulmonary arterial hypertension. Nat Commun. 2019;10:3551. 10.1038/s41467-019-11500-6. 31391533 10.1038/s41467-019-11500-6 PMC6686016

[CR33] Ma C, Wang X, He S, Zhang L, Bai J, Qu L, Qi J, Zheng X, Zhu X, Mei J, Guan X, Yuan H, Zhu D. Ubiquitinated AIF is a major mediator of hypoxia-induced mitochondrial dysfunction and pulmonary artery smooth muscle cell proliferation. Cell Biosci. 2022. 10.1186/s13578-022-00744-3. 35090552 10.1186/s13578-022-00744-3 PMC8796423

[CR34] Matsufuji S, Kitajima Y, Higure K, Kimura N, Maeda S, Yamada K, Ito K, Tanaka T, Kai K, Noshiro H. A HIF-1α inhibitor combined with palmitic acid and L-carnitine treatment can prevent the fat metabolic reprogramming under hypoxia and induce apoptosis in hepatocellular carcinoma cells. Cancer Metab. 2023. 10.1186/s40170-023-00328-w. 38066600 10.1186/s40170-023-00328-w PMC10709876

[CR35] Mistry JJ, Hellmich C, Moore JA, Jibril A, Macaulay I, Moreno-Gonzalez M, Di Palma F, Beraza N, Bowles KM, Rushworth SA. Free fatty-acid transport via CD36 drives β-oxidation-mediated hematopoietic stem cell response to infection. Nat Commun. 2021. 10.1038/s41467-021-27460-9. 34880245 10.1038/s41467-021-27460-9 PMC8655073

[CR36] Niu H, Ren X, Tan E, Wan X, Wang Y, Shi H, Hou Y, Wang L. CD36 deletion ameliorates diabetic kidney disease by restoring fatty acid oxidation and improving mitochondrial function. Ren Fail. 2023. 10.1080/0886022x.2023.2292753. 38097943 10.1080/0886022X.2023.2292753 PMC10732185

[CR37] Ornatowski W, Lu Q, Yegambaram M, Garcia AE, Zemskov EA, Maltepe E, Fineman JR, Wang T, Black SM. Complex interplay between autophagy and oxidative stress in the development of pulmonary disease. Redox Biol. 2020;36: 101679. 10.1016/j.redox.2020.101679. 32818797 10.1016/j.redox.2020.101679 PMC7451718

[CR38] Pan J, Fan Z, Wang Z, Dai Q, Xiang Z, Yuan F, Yan M, Zhu Z, Liu B, Li C. CD36 mediates palmitate acid-induced metastasis of gastric cancer via AKT/GSK-3β/β-catenin pathway. J Exp Clin Cancer Res. 2019. 10.1186/s13046-019-1049-7. 30717785 10.1186/s13046-019-1049-7 PMC6360779

[CR39] Pitre T, Su J, Cui S, Scanlan R, Chiang C, Husnudinov R, Khalid MF, Khan N, Leung G, Mikhail D, Saadat P, Shahid S, Mah J, Mielniczuk L, Zeraatkar D, Mehta S. Medications for the treatment of pulmonary arterial hypertension: a systematic review and network meta-analysis. Eur Respir Rev. 2022. 10.1183/16000617.0036-2022. 35948391 10.1183/16000617.0036-2022 PMC9724821

[CR40] Poole LP, Bock-Hughes A, Berardi DE, Macleod KF. ULK1 promotes mitophagy via phosphorylation and stabilization of BNIP3. Sci Rep. 2021. 10.1038/s41598-021-00170-4. 34654847 10.1038/s41598-021-00170-4 PMC8519931

[CR41] Romanoski CE, Qi X, Sangam S, Vanderpool RR, Stearman RS, Conklin A, Gonzalez-Garay M, Rischard F, Ayon RJ, Wang J, Simonson T, Babicheva A, Shi Y, Tang H, Makino A, Kanthi Y, Geraci MW, Garcia JGN, Yuan JX, Desai AA. Transcriptomic profiles in pulmonary arterial hypertension associate with disease severity and identify novel candidate genes. Pulm Circ. 2020;10:2045894020968531. 10.1177/2045894020968531. 33343881 10.1177/2045894020968531 PMC7727059

[CR42] Samovski D, Jacome-Sosa M, Abumrad NA. Fatty acid transport and signaling: mechanisms and physiological implications. Annu Rev Physiol. 2023;85:317–37. 10.1146/annurev-physiol-032122-030352. 36347219 10.1146/annurev-physiol-032122-030352 PMC13221695

[CR43] Shah AJ, Vorla M, Kalra DK. Molecular pathways in pulmonary arterial hypertension. Int J Mol Sci. 2022. 10.3390/ijms231710001. 36077398 10.3390/ijms231710001 PMC9456336

[CR44] Shimoda LA. Cellular pathways promoting pulmonary vascular remodeling by hypoxia. Physiology (Bethesda). 2020;35:222–33. 32490752 10.1152/physiol.00039.2019 PMC7474258

[CR45] Su L, Zhang J, Gomez H, Kellum JA, Peng Z. Mitochondria ROS and mitophagy in acute kidney injury. Autophagy. 2022;19:401–14. 10.1080/15548627.2022.2084862. 35678504 10.1080/15548627.2022.2084862 PMC9851232

[CR46] Talati M, Hemnes A. Fatty acid metabolism in pulmonary arterial hypertension: role in right ventricular dysfunction and hypertrophy. Pulm Circ. 2015;5:269–78. 10.1086/681227. 26064451 10.1086/681227 PMC4449237

[CR47] Tian XY, Ma S, Tse G, Wong WT, Huang Y. Uncoupling protein 2 in cardiovascular health and disease. Front Physiol. 2018. 10.3389/fphys.2018.01060. 30116205 10.3389/fphys.2018.01060 PMC6082951

[CR48] Tirpe AA, Gulei D, Ciortea SM, Crivii C, Berindan-Neagoe I. Hypoxia: overview on hypoxia-mediated mechanisms with a focus on the role of HIF genes. Int J Mol Sci. 2019. 10.3390/ijms20246140. 31817513 10.3390/ijms20246140 PMC6941045

[CR49] Veith C, Schermuly RT, Brandes RP, Weissmann N. Molecular mechanisms of hypoxia-inducible factor-induced pulmonary arterial smooth muscle cell alterations in pulmonary hypertension. J Physiol. 2016;594:1167–77. 10.1113/JP270689. 26228924 10.1113/JP270689 PMC4771790

[CR50] Wang J, Li Y. CD36 tango in cancer: signaling pathways and functions. Theranostics. 2019;9:4893–908. 10.7150/thno.36037. 31410189 10.7150/thno.36037 PMC6691380

[CR51] Wilkins MR, Ghofrani H-A, Weissmann N, Aldashev A, Zhao L. Pathophysiology and treatment of high-altitude pulmonary vascular disease. Circulation. 2015;131:582–90. 10.1161/circulationaha.114.006977. 25666980 10.1161/CIRCULATIONAHA.114.006977

[CR52] Xie Y, Shi X, Sheng K, Han G, Li W, Zhao Q, Jiang B, Feng J, Li J, Gu Y. PI3K/Akt signaling transduction pathway, erythropoiesis and glycolysis in hypoxia (Review). Mol Med Rep. 2019;19:783–91. 10.3892/mmr.2018.9713. 30535469 10.3892/mmr.2018.9713 PMC6323245

[CR53] Xu W, Janocha AJ, Erzurum SC. Metabolism in pulmonary hypertension. Annu Rev Physiol. 2021;83:551–76. 10.1146/annurev-physiol-031620-123956. 33566674 10.1146/annurev-physiol-031620-123956 PMC8597719

[CR54] Yang L, Tian J, Wang J, Zeng J, Wang T, Lin B, Linneman J, Li L, Niu Y, Gou D, Zhang Y. The protective role of EP300 in monocrotaline-induced pulmonary hypertension. Front Cardiovasc Med. 2023. 10.3389/fcvm.2023.1037217. 36910531 10.3389/fcvm.2023.1037217 PMC9992637

[CR55] Zeng S, Wu F, Chen M, Li Y, You M, Zhang Y, Yang P, Wei L, Ruan XZ, Zhao L, Chen Y. Inhibition of fatty acid translocase (FAT/CD36) palmitoylation enhances hepatic fatty acid β-oxidation by increasing its localization to mitochondria and interaction with long-chain acyl-CoA synthetase 1. Antioxid Redox Signal. 2022;36:1081–100. 35044230 10.1089/ars.2021.0157

[CR56] Zhang T, Liu Q, Gao W, Sehgal SA, Wu H. The multifaceted regulation of mitophagy by endogenous metabolites. Autophagy. 2021;18:1216–39. 10.1080/15548627.2021.1975914. 34583624 10.1080/15548627.2021.1975914 PMC9225590

[CR57] Zhou T, Chang L, Luo Y, Zhou Y, Zhang J. Mst1 inhibition attenuates non-alcoholic fatty liver disease via reversing Parkin-related mitophagy. Redox Biol. 2019. 10.1016/j.redox.2019.101120. 30708325 10.1016/j.redox.2019.101120 PMC6357900

[CR58] Zhu J, Zhao L, Hu Y, Cui G, Luo A, Bao C, Han Y, Zhou T, Lu W, Wang J, Black SM, Tang H. Hypoxia-inducible factor 2-alpha mediated gene sets differentiate pulmonary arterial hypertension. Front Cell Dev Biol. 2021;9: 701247. 10.3389/fcell.2021.701247. 34422822 10.3389/fcell.2021.701247 PMC8375387

[CR59] Zhu P, Wang X, Wu Q, Zhu J, Que Y, Wang Y, Ding Y, Yang Y, Jin J, Zhang X, Xu Q, Yong Q, Chang C, Xu G, Du Y. BCAP31 alleviates lipopolysaccharide-mediated acute lung injury via induction of PINK1/Parkin in alveolar epithelial type II cell. Research. 2024;7:0498. 10.34133/research.0498. 39381793 10.34133/research.0498 PMC11458857

