# Supplementary figures and images for: PI3K p85α/HIF-1α accelerates the development of pulmonary arterial hypertension by regulating fatty acid uptake and mitophagy
Source: Mol Med. 2024 Nov 11;30:208. doi: 10.1186/s10020-024-00975-9 (PMC11552344; doi:10.1186/s10020-024-00975-9)

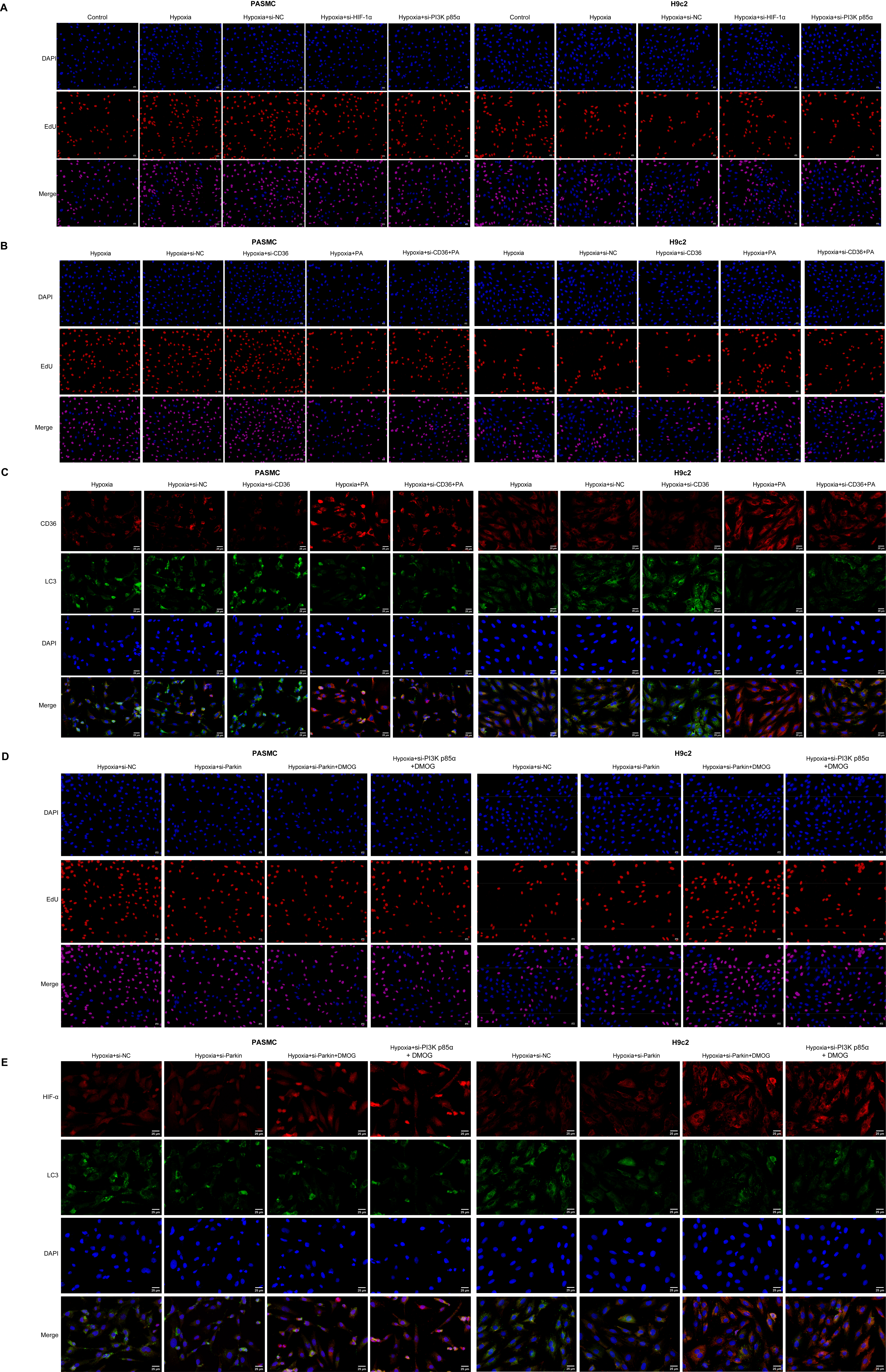

Supplement: Supplementary file 1 — Additional file 1: Figure S1. Representative fluorescence images. (A) Representative fluorescence images of DAPI (blue) and EdU (red) labeling in 2D. (B) Representative fluorescence images of DAPI (blue) and EdU (red) labeling in 3E. (C) Representative fluorescence images of DAPI (blue), CD36 (red), and LC3 (green) labeling in 3K. (D) Representative fluorescence images of DAPI (blue) and EdU (red) labeling in 4C. (E) Representative fluorescence images of DAPI (blue), HIF-1α (red), and LC3 (green) labeling in 4G. [file 10020_2024_975_MOESM1_ESM.jpg]

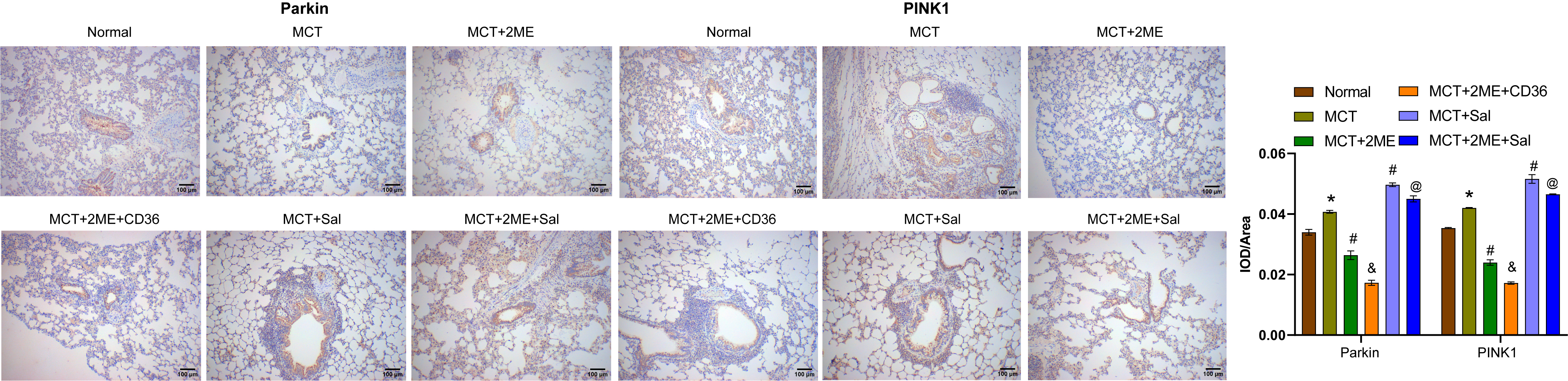

Supplement: Supplementary file 2 — Additional file 2: Figure S2. Representative immunohistochemistry images and statistical analysis of Parkin and PINK1 in lung tissue. *P < 0.05 vs. Normal. #P < 0.05 vs. PAH. &P < 0.05 vs. PAH+2ME. @P < 0.05 vs. PAH+Sal. n = 6. The statistical test was performed using one-way ANOVA, followed by Tukey’s post hoc test. [file 10020_2024_975_MOESM2_ESM.jpg]
