# Supplementary material for: PI3K p85α/HIF-1α accelerates the development of pulmonary arterial hypertension by regulating fatty acid uptake and mitophagy
Source: Mol Med. 2024 Nov 11;30:208. doi: 10.1186/s10020-024-00975-9 (PMC11552344; doi:10.1186/s10020-024-00975-9)
Supplement: Supplementary file 3 — Additional file 3. [file 10020_2024_975_MOESM3_ESM.docx]

**Supplementary Table 1. Patients clinical and pathological characteristics.**

| ID | Sex | Age  (years) | Height  (cm) | Body weight  (kg) | WTO-FC | Hemoglobin  (g/dL) | BNP (pg/mL) | mPAP  (mmHg) | PCWP  (mmHg) | PVR  (WU) | LAD  (mm) | LVEF  (%) | RAP  (mmHg) | RVP  (mmHg) | CO  (L/min) | |
| --- | --- | --- | --- | --- | --- | --- | --- | --- | --- | --- | --- | --- | --- | --- | --- | --- |
| PAH-01 | female | 23 | 158 | 50 | 2 | 146 | 149.5 | 62 | 9 | 13.66 | 29 | 55 | 10 | 42 | 3.88 |  |
| PAH-02 | female | 64 | 156 | 45 | 3 | 148 | 2644 | 50 | 9 | 11.77 | 34 | 70 | 7 | 40 | 3.49 |  |
| PAH-03 | male | 60 | 165 | 64 | 3 | 136 | 2589 | 36 | 13 | 8.17 | 48 | 43 | 14 | 23 | 2.83 |  |
| PAH-04 | male | 67 | 185 | 62.5 | 3 | 149 | 1123 | 26 | 3 | 2.92 | 34 | 66 | 4 | 24 | 7.89 |  |
| PAH-05 | female | 31 | 161 | 53 | 2 | 152 | 628.36 | 69 | 14 | 12.53 | 30 | 74 | 13 | 47 | 4.39 |  |
| PAH-06 | female | 28 | 155 | 35 | 2 | 191 | 265 | 59 | 10 | 27.2 | 14 | 53 | 0 | 31 | 1.8 |  |
| PAH-07 | male | 61 | 158 | 49 | 3 | 141 | 4839 | 33 | 7 | 9.813 | 37 | 47 | 9 | 20 | 2.65 |  |
| PAH-08 | female | 46 | 150 | 43 | 2 | 133 | 286 | 56 | 5 | 18.82 | 39 | 44 | 6 | 38 | 2.71 |  |
| PAH-09 | female | 33 | 148 | 54 | 2 | 116 | 132 | 41 | 3 | 3.8 | 32 | 70 | 1 | 30 | 8.6 |  |
| PAH-10 | female | 48 | 153 | 51 | 2 | 162 | 2461.4 | 66 | 9 | 28.93 | 34 | 55 | 8 | 42 | 1.97 |  |
| PAH-11 | female | 55 | 169 | 65.5 | 3 | 127 | 1927 | 40 | 4 | 3.11 | 42 | 61 | 24 | 29 | 3.38 |  |
| PAH-12 | female | 28 | 157 | 55 | 2 | 127 | 100 | 67 | 7 | 18.35 | 33 | 50 | 9 | 43 | 3.27 |  |
| PAH-13 | female | 58 | 160 | 58.5 | 2 | 133 | 50 | 27 | 6 | 5.02 | 37 | 67 | 10 | 22 | 4.18 |  |
| PAH-14 | female | 48 | 153 | 38 | 2 | 145 | 359.9 | 52 | 14 | 22.75 | 42 | 52 | 3 | 58 | 1.67 |  |
| PAH-15 | female | 64 | 160 | 55 | 2 | 119 | 75.3 | 26 | 10 | 3.6 | 45 | 61 | 4 | 13 | 4.4 |  |
| PAH-16 | female | 36 | 158 | 52 | 3 | 139 | 1050 | 79 | 2 | 21.1 | 39 | 62 | 6 | 43 | 3.65 |  |
| PAH-17 | female | 31 | 160 | 41 | 2 | 128 | 653.5 | 63 | 8 | 19.64 | 29 | 52 | 9 | 40 | 2.8 |  |
| PAH-18 | female | 53 | 160 | 50 | 2 | 135 | 5846 | 40 | 3 | 22.6 | 50 | 37 | 15 | 26 | 1.77 |  |
| PAH-19 | female | 31 | 170 | 81 | 2 | 120 | 291 | 51 | 14.7 | 12.53 | 33 | 65 | 15 | 35 | 3.8 |  |
| PAH-20 | male | 43 | 163 | 53 | 3 | 77 | 2422.05 | 52 | 11.6 | 11.6 | 30 | 64 | 3 | 32 | 4.48 |  |
| Normal-01 | female | 38 | / | / | / | / | / | / | / | / | / | / | / | / | / |  |
| Normal-02 | female | 38 | / | / | / | / | / | / | / | / | / | / | / | / | / |  |
| Normal-03 | male | 44 | / | / | / | / | / | / | / | / | / | / | / | / | / |  |
| Normal-04 | female | 43 | / | / | / | / | / | / | / | / | / | / | / | / | / |  |
| Normal-05 | female | 43 | / | / | / | / | / | / | / | / | / | / | / | / | / |  |
| Normal-06 | male | 44 | / | / | / | / | / | / | / | / | / | / | / | / | / |  |
| Normal-07 | male | 40 | / | / | / | / | / | / | / | / | / | / | / | / | / |  |
| Normal-08 | female | 36 | / | / | / | / | / | / | / | / | / | / | / | / | / |  |
| Normal-09 | male | 35 | / | / | / | / | / | / | / | / | / | / | / | / | / |  |
| Normal-10 | female | 38 | / | / | / | / | / | / | / | / | / | / | / | / | / |  |
| Normal-11 | male | 32 | / | / | / | / | / | / | / | / | / | / | / | / | / |  |
| Normal-12 | female | 39 | / | / | / | / | / | / | / | / | / | / | / | / | / |  |
| Normal-13 | female | 41 | / | / | / | / | / | / | / | / | / | / | / | / | / |  |
| Normal-14 | male | 31 | / | / | / | / | / | / | / | / | / | / | / | / | / |  |
| Normal-15 | female | 41 | / | / | / | / | / | / | / | / | / | / | / | / | / |  |
| Normal-16 | female | 41 | / | / | / | / | / | / | / | / | / | / | / | / | / |  |
| Normal-17 | male | 40 | / | / | / | / | / | / | / | / | / | / | / | / | / |  |
| Normal-18 | female | 30 | / | / | / | / | / | / | / | / | / | / | / | / | / |  |
| Normal-19 | male | 31 | / | / | / | / | / | / | / | / | / | / | / | / | / |  |
| Normal-20 | female | 24 | / | / | / | / | / | / | / | / | / | / | / | / | / |  |

Note: PAH, pulmonary arterial hypertension; WHO-FC, World Health Organization functional class; BNP, brain natriuretic peptide; mPAP, mean pulmonary arterial pressure; PCWP, pulmonary capillary wedge pressure; PVR, pulmonary vascular resistance; WU, wood unit; LAD, left atrial diameter; LVEF, left ventricular ejection fraction; RAP, right atrium pressure; RVP, right ventricular pressure; CO, cardiac output.
