# Supplementary material for: PI3K p85α/HIF-1α accelerates the development of pulmonary arterial hypertension by regulating fatty acid uptake and mitophagy
Source: Mol Med. 2024 Nov 11;30:208. doi: 10.1186/s10020-024-00975-9 (PMC11552344; doi:10.1186/s10020-024-00975-9)
Supplement: Supplementary file 4 — Additional file 4. [file 10020_2024_975_MOESM4_ESM.docx]

**Supplementary Table 2. Primer sequences used in this study.**

| **Gene** | **Sequences (5'-3')** |
| --- | --- |
| H-HIF-1α | Forward: 5'- TGGTATTATTCAGCACGACT -3' |
|  | Reverse: 5'- GCCAGCAAAGTTAAAGCATC -3' |
| H-PI3K | Forward: 5'- TGCGTCTACTAAAATGCATGG -3' |
|  | Reverse: 5'- AACTGAAGGTTAATGGGTCA -3' |
| H-Parkin | Forward: 5'- GTGTTTGTCAGGTTCAACTCCA -3' |
|  | Reverse: 5'- GAAAATCACACGCAACTGGTC -3' |
| H-PINK1 | Forward: 5'- TGACCTTTGCCCCTAACACGAG -3' |
|  | Reverse: 5'- GTAACTGAACGTGCTGACCCAT -3' |
| H-CD36 | Forward: 5'- AAAATGTAACCCAGGACG -3' |
|  | Reverse: 5'- GTGTCGATTATGGCAACT -3' |
| H-UCP2 | Forward: 5'- CCCCGAAGCCTCTACAATGG -3' |
|  | Reverse: 5'- CTGAGCTTGGAATCGGACCTT -3' |
| H-MnSOD | Forward: 5'- CGTCAAAGTCATTCTCCGTCCA -3' |
|  | Reverse: 5'- AAAACCCCATCTGCACATCACC -3' |
| H-ULK1 | Forward: 5'- AGCACGATTTGGAGGTCGC -3' |
|  | Reverse: 5'- GCCACGATGTTTTCATGTTTCA -3' |
| H-BNIP3L | Forward: 5'- ATGTCGTCCCACCTAGTCGAG -3' |
|  | Reverse: 5'- TGAGGATGGTACGTGTTCCAG -3' |
| H-FUNDC1 | Forward: 5'- CCACAGTTCGGGACCTATGG -3' |
|  | Reverse: 5'- AGCCACTATGACTAGCAATCTGA -3' |
| H-GAPDH | Forward: 5'- ACAGCCTCAAGATCATCAGC -3' |
|  | Reverse: 5'- GGTCATGAGTCCTTCCACGAT -3' |
| R-HIF-1α | Forward: 5'- ACGATTGTGAAGTTAATGCTCCC -3' |
|  | Reverse: 5'- AACCAACAGAAACGAAACCCC -3' |
| R-PI3K | Forward: 5'- AGCCACAGATCCACTTAACCC -3' |
|  | Reverse: 5'- CTTGCTGTCCCCACTTTACTGA -3' |
| R-Parkin | Forward: 5'- TCCAATGTAACCACCGCCACG -3' |
|  | Reverse: 5'- AGTAGCCAAGTTGAGCGTCGTG -3' |
| R-PINK1 | Forward: 5'- GTGTCTGACCCACTGGACAC -3' |
|  | Reverse: 5'- CTGCTCCCTTTGAGACGACA -3' |
| R-CD36 | Forward: 5'- TGAGCCTTCACTGTCTGTTGGAAC -3' |
|  | Reverse: 5'- AGGCTGTTGAGCACACCTTGAAC -3' |
| R-UCP2 | Forward: 5'- CAATGTTGCCCGAAATGC -3' |
|  | Reverse: 5'- CAAGGGAGGTCGTCTGTC -3' |
| R-MnSOD | Forward: 5'- CGTCAAAGTCATTCTCCGTCCA -3' |
|  | Reverse: 5'- AAAACCCCATCTGCACATCACC -3' |
| R-ULK1 | Forward: 5'- ACACACCCTCTCCCCAAGTG -3' |
|  | Reverse: 5'- GGTTCGTGGAGAGTGCTCAG -3' |
| R-BNIP3L | Forward: 5'- CGCCCTGAATGGGTAGGAGA -3' |
|  | Reverse: 5'- TGATGTTAAACCTGCGAGCAT -3' |
| R-FUNDC1 | Forward: 5'- GCGAGCAAATAAGGCAGCAC -3' |
|  | Reverse: 5'- AGCAAAAAGCCTCCCACGAA -3' |
| R-GAPDH | Forward: 5'- ACAGCAACAGGGTGGTGGAC -3' |
|  | Reverse: 5'- TTTGAGGGTGCAGCGAACTT -3' |
