# Supplementary material for: PI3K p85α/HIF-1α accelerates the development of pulmonary arterial hypertension by regulating fatty acid uptake and mitophagy
Source: Mol Med. 2024 Nov 11;30:208. doi: 10.1186/s10020-024-00975-9 (PMC11552344; doi:10.1186/s10020-024-00975-9)
Supplement: Supplementary file 5 — Additional file 5. [file 10020_2024_975_MOESM5_ESM.docx]

**Supplementary Table 3. Antibody information.**

| **Indicator** | **Dilution** | **Origin** | **Catalog** | **Manufacturer** |
| --- | --- | --- | --- | --- |
| HIF-1α | 1: 1000 | Mouse | NB100-105 | Novus Biologicals, Centennial, CO, USA |
| PI3K p85α | 1: 1000 | Rabbit | ab191606 | Abcam |
| Parkin | 1: 2000 | Rabbit | 14060-1-AP | Proteintech |
| PINK1 | 1: 600 | Rabbit | 23274-1-AP | Proteintech |
| CD36 | 1: 1000 | Rabbit | NB400-144 | Novus Biologicals |
| LC3 | 1: 2000 | Rabbit | 14600-1-AP | Proteintech |
| ATG7 | 1: 800 | Rabbit | 10088-2-AP | Proteintech |
| p62 | 1: 3000 | Rabbit | ab155686 | Abcam |
| PCNA | 1: 5000 | Rabbit | 10205-2-AP | Proteintech |
| UCP2 | 1: 1000 | Rabbit | 11081-1-AP | Proteintech |
| MnSOD | 1: 10000 | Rabbit | 24127-1-AP | Proteintech |
| ULK1 | 1 µg/mL | Rabbit | ab167139 | Abcam |
| BINP3L | 1: 1000 | Rabbit | 12986-1-AP | Proteintech |
| FUNDC1 | 1: 8000 | Rabbit | 28519-1-AP | Proteintech |
| GAPDH | 1: 5000 | Rabbit | 10494-1-AP | Proteintech |
| PCNA | 1: 5000 | Rabbit | 10205-2-AP | Proteintech |
| HRP goat anti-rabbit IgG | 1: 6000 | Rabbit | SA00001-2 | Proteintech |
| HRP goat anti-mouse IgG | 1: 5000 | Mouse | SA00001-1 | Proteintech |
